# Evolutionary interplay between sister cytochrome P450 genes shapes plasticity in plant metabolism

**DOI:** 10.1038/ncomms13026

**Published:** 2016-10-07

**Authors:** Zhenhua Liu, Raquel Tavares, Evan S. Forsythe, François André, Raphaël Lugan, Gabriella Jonasson, Stéphanie Boutet-Mercey, Takayuki Tohge, Mark A. Beilstein, Danièle Werck-Reichhart, Hugues Renault

**Affiliations:** 1 Institute of Plant Molecular Biology, CNRS, University of Strasbourg, 12 rue du Général Zimmer, Strasbourg 67084 France; 2 Université Lyon 1, CNRS, UMR 5558, Laboratoire de Biométrie et Biologie Évolutive, 16 rue Raphael Dubois, 69622 Villeurbanne Cedex, France; 3 School of Plant Sciences, University of Arizona, Tucson, Arizona 85721, USA; 4 iBiTec-S/SB^2^SM, UMR 9198 CNRS, University Paris Sud, CEA Saclay, 91191 Gif-sur-Yvette, France; 5 Institut Jean-Pierre Bourgin, UMR 1318 INRA-AgroParisTech, Saclay Plant Sciences RD10, 78026 Versailles, France; 6 Max-Planck-Institute of Molecular Plant Physiology, Department of Molecular Physiology, 14476 Potsdam-Golm, Germany; 7 University of Strasbourg Institute for Advanced Study, 67000 Strasbourg, France; 8 Freiburg Institute for Advanced Studies, University of Freiburg, 79104 Freiburg, Germany

## Abstract

Expansion of the cytochrome P450 gene family is often proposed to have a critical role in the evolution of metabolic complexity, in particular in microorganisms, insects and plants. However, the molecular mechanisms underlying the evolution of this complexity are poorly understood. Here we describe the evolutionary history of a plant P450 retrogene, which emerged and underwent fixation in the common ancestor of Brassicales, before undergoing tandem duplication in the ancestor of Brassicaceae. Duplication leads first to gain of dual functions in one of the copies. Both sister genes are retained through subsequent speciation but eventually return to a single copy in two of three diverging lineages. In the lineage in which both copies are maintained, the ancestral functions are split between paralogs and a novel function arises in the copy under relaxed selection. Our work illustrates how retrotransposition and gene duplication can favour the emergence of novel metabolic functions.

## Abstract (web summary)

Genes in the cytochrome P450 family have evolved a wide range of functions. Here, Liu *et al.* reconstruct the evolution of the P450 genes *CYP98A8* and *CYP98A9* in the Brassicales, revealing a complex history of retrotransposition, tandem duplication and neofunctionalization, followed by subfunctionalization or gene loss in specific lineages.

## Graphical abstract

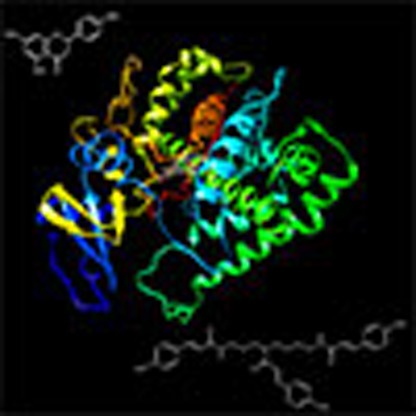

Evolution of new protein functions is often initiated by gene duplication, but most duplicates are discarded by purifying selection^1^,^2^,^3^,^4^,^5^. The retention of new copies may be explained by either neofunctionalization or subfunctionalization^1^,^6^,^7^, with several models for subfunctionalization proposed^5^,^7^,^8^,^9^,^10^,^11^. Functional innovation may result from an expansion of a previous biochemical activity or from a change in the biochemical properties of the protein^12^,^13^. Acquisition of an additional biological activity can then lead to a pleiotropic protein. Multiple analyses have shown that the evolutionary scenario after gene duplication may be more complex, implicating several of these mechanisms, acting one after another^14^,^15^,^16^, as has been suggested by theory^17^. However, experimental evidence of the models and their interplay is often difficult to obtain. We describe here the evolutionary history of a plant retrogene^18^ that stemmed at an early step of Brassicales evolution.

The parent gene, represented by *CYP98A3* in *Arabidopsis thaliana* (family Brassicaceae, order Brassicales), controls a major branch point in the plant phenolic pathway^19^,^20^. It is broadly expressed in plants, especially in vascular tissues, and encodes the phenolic ring *meta*-hydroxylase of lignin precursors. This enzyme evolved in land plants around 400 million years ago (Ma) to provide metabolites essential for protection against desiccation and ultraviolet radiation, water conduction and erect growth^21^. Due to its gate-keeping role and the high flux in the lignin pathway, *CYP98A3* and its orthologs are under strong purifying selection^18^. Only a few lineage-specific duplications are inferred in phylogenies of *CYP98A3* across land plants. One such duplication occurred via transposition within Brassicales and was followed by a tandem duplication giving rise to *CYP98A8* and *CYP98A9* genes leading to the formation of *N*^*1*^*,N*^*5*^-di(hydroxyferuloyl)-*N*^*10*^-sinapoyl-spermidine, a major pollen coat and wall constituent in *A. thaliana*^18^,^22^. Available data indicate that both CYP98A8 and CYP98A9 catalysed the first *meta*-hydroxylation of the phenolic ring of pollen constituents (3′-hydroxylation of *N*^*1*^*,N*^*5*^*,N*^*10*^-tri-coumaroyl-spermidine) and that CYP98A8 also catalysed the second (5′-hydroxylation of *N*^*1*^*,N*^*5*^*,N*^*10*^-tri-feruloyl-spermidine). The recent emergence of this pathway and the sequencing of a representative set of Brassicales genomes offers the opportunity to determine the evolutionary process leading to these new P450 functions.

We show here that the founding retroposition leading to the CYP98A8 and CYP98A9 common ancestor occurred before the divergence of Cleomaceae and was followed by a duplication before the divergence of the core Brassicaceae lineages. We demonstrate that the fate and function of the resulting paralogs differ between the Brassicaceae lineages, with either loss of one of the paralogs, the other maintaining a dual function in lineage II and III, or conservation of the two gene copies in lineage I, with subfunctionalization and gain of an additional activity in flavonoid metabolism by one of the duplicates.

## Results

### Evolutionary history of the CYP98 family in Brassicales

**Figure 1:**
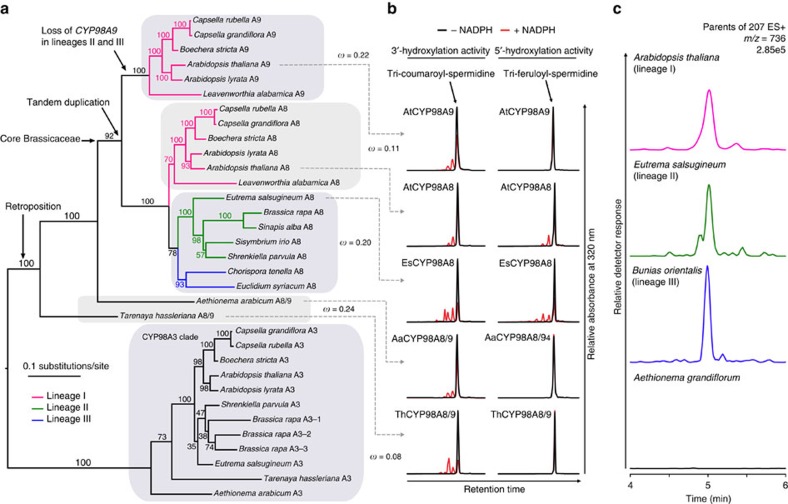
Evolutionary history of the CYP98A8/9 clade in Brassicales/Brassicaceae. (**a**) Annotated protein maximum likelihood tree illustrating the phylogenetic relationships in the CYP98 family in Brassicales. The CYP98A3 clade is the parent to CYP98A8/9s. Evolution regimes (*ω*=*d*_N_/*d*_S_) computed with codeml Branch model and acting on different phylogenetic groups are indicated. Branch support in the tree was calculated from 1,000 bootstraps replicates. (**b**) *In vitro* assay of CYP98A8/A9 enzymes activities. Tri-coumaroyl-spermidine and tri-feruloyl-spermidine were used to assess the 3′- and 5′-hydroxylase activities of yeast-expressed proteins. CYP98A8/A9 from all species have 3′-hydroxylase activity. Only CYP98A8 from Brassicaceae have 5′-hydroxylase activity. (**c**) Search for sinapoylated (*m/z* 207) phenolamides by parent ion scan analysis of flower buds demonstrates the absence *in vivo* of the tri-hydroxylated phenolamide di-(hydroxyferuloyl)-sinapoyl-spermidine (*m/z* 736) in *A. grandiflorum*, while it is present in Brassicaceae from lineages I, II and III. Colour of each profile is according to the lineage colour code in **a**.

A large set of genomes in Brassicales has now been sequenced, allowing the reconstruction of CYP98A8 and CYP98A9 evolutionary history (Fig. 1a; Supplementary Fig. 1 and Supplementary Table 1). The reconstructed CYP98 phylogeny first shows that a *CYP98A8/9* homolog is found in some, but not all Brassicales. For example, *Tarenaya hassleriana*, belonging to Cleomaceae, the sister family that split from Brassicaceae around 70 Ma^23^,^24^, contains a *CYP98A8/9* copy, but the more early diverging *Carica papaya* (Caricaceae; 100 MY divergence with Brassicaceae) does not. This dates the *CYP98A8/9* founding retroposition between 70 and 100 Ma^25^. The tandem duplication generating the *CYP98A8* and *CYP98A9* paralogs occurred after the divergence of the genus *Aethionema*, predating the divergence of the major lineages I, II and III of core Brassicaceae (Fig. 1a; ref. 23). The duplication pattern indicates that lineages II and III subsequently lost *CYP98A9*, while lineage I retained it. A *CYP98A8* ortholog is present in all available Brassicaceae genomes, which include lineage I and lineage II species^23^, but *CYP98A9* is found only in lineage I (Fig. 1a).

In the absence of sequenced lineage III species, we designed degenerate primers to amplify *CYP98A8/9* homologs from the lineage III species *Chorispora tenella* and *Euclidium syriacum*. A *CYP98A8* orthologous sequence could be retrieved from both species (Supplementary Fig. 2), but no *CYP98A9.* However, BLAST search of the *CYP98A8* flanking regions, respectively, found one and four correctly ordered remnant *CYP98A9* fragments in *C. tenella* and *E. syriacum*, respectively (Supplementary Fig. 2), indicating that *CYP98A9* had been present and was subsequently lost from the genome. The presence of a fragmented CYP98A9 confirms the duplication history inferred from the gene tree. Thus, the tandem duplication event that gave birth to *CYP98A9* predates the split between sister lineages I, II and III, around 45 Ma, and *CYP98A9* is retained only in lineage I.

### Activity of the CYP98A8/9 proteins *in vivo* and *in vitro*

We then assessed CYP98 enzyme activity in each lineage using two complementary approaches. One was the yeast-expression of representative *CYP98A8/A9* genes for *in vitro* assay of catalytic activity (Fig. 1b). The second employed metabolic profiling of flower buds from members of each Brassicaceae lineage and a representative of the earliest diverging branch in the family (that is, the genus *Aethionema*) (Fig. 1c). *In vitro* assays revealed that: (1) the CYP98A8/9 protein from *Adenium arabicum* and *T. hassleriana* catalysed only 3′-hydroxylation of tri-coumaroyl-spermidine; (2) the radiation of core Brassicaceae was preceded by acquisition of the 5′-hydroxylase activity, shown by the ability of CYP98A8 from *Eutrema salsugineum* (lineage II) and *A. thaliana* (lineage I) to catalyse both 3′-hydroxylation of tri-coumaroyl-spermidine and 5′-hydroxylation of tri-feruloyl-spermidine; (3) *A. thaliana* CYP98A9 has 3′- but not 5′-hydroxylase activity, similar to *A. arabicum* and *T. hassleriana*. Taken together, our *in vitro* data suggest that 5′-hydroxylase activity evolved in CYP98A8, post duplication.

Phenolic profiling supported the results from the enzyme assays. The 5′-hydroxylated phenolamide di-(hydroxyferuloyl)-sinapoyl-spermidine was absent from the flowers of *Aethionema grandiflorum*, but present as at least two different isomers in core Brassicaceae (Fig. 1c; Supplementary Fig. 3). Hence, we inferred that the single *CYP98A8/9* copy in the ancestor of both Brassicaceae and Cleomaceae lacked 5′-hydroxylase activity. Moreover, the single *CYP98A8* copy present in lineages II and III performed both 3′- and 5′-hydroxylations, *in vitro* and *in vivo*. This finding is also supported for lineage I species by the ability of *A. thaliana* CYP98A8 to catalyse both reactions *in vitro*. Both sets of analyses thus concurred to indicate that the acquisition of 5′-hydroxylation occurred in the ancestor of core Brassicaceae. However, whether this acquisition predated, or was subsequent to, the gene duplication that gave rise to *CYP98A9* remained an open question.

**Figure 2:**
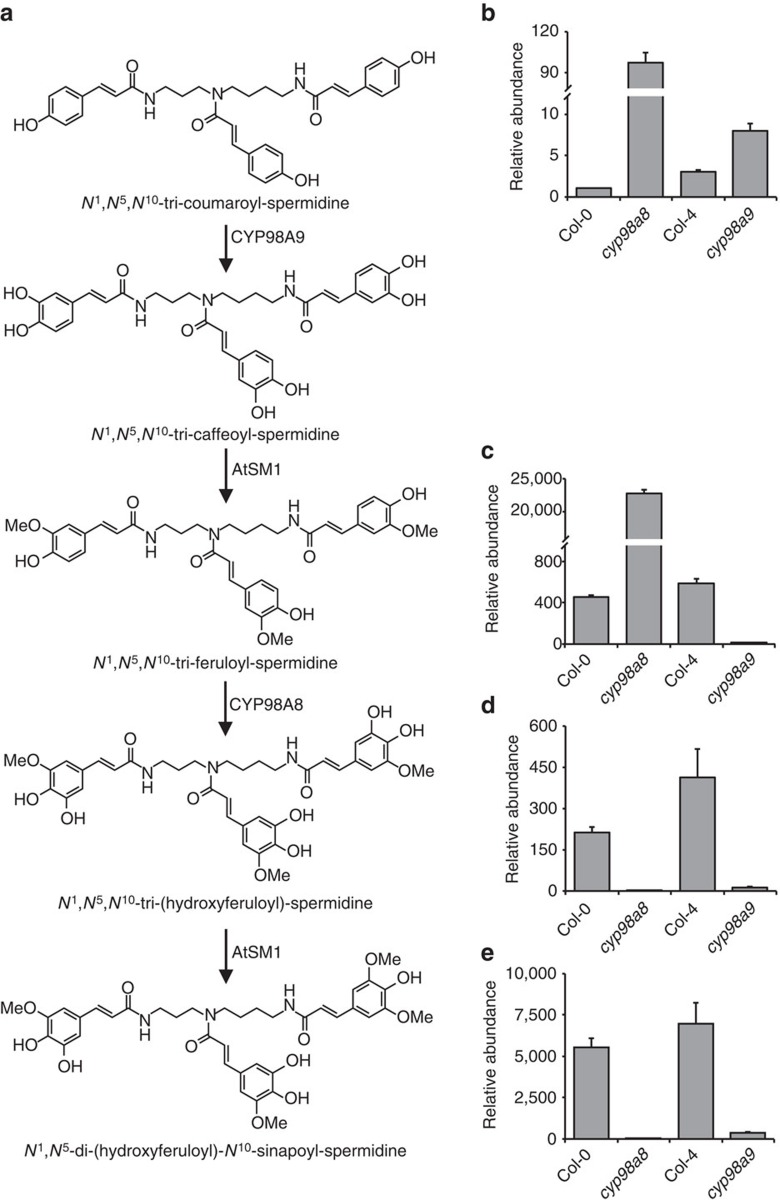
Functional specialization of CYP98A8 and CYP98A9 *in vivo*. (**a**) The anther-specific phenolamide pathway as previously delineated in *A. thaliana*. (**b**–**e**) Targeted metabolic profiling in *A. thaliana* flower buds shows that phenolamide intermediates downstream of *N*^*1*^*,N*^*5*^*,N*^*10*^-tri-coumaroyl-spermidine are absent from the *cyp98a9* mutant, indicating that CYP98A8 does not perform the 3′-hydroxylation *in vivo*: (**b**) *N*^*1*^*,N*^*5*^*,N*^*10*^-tri-coumaroyl-spermidine, (**c**) *N*^*1*^*,N*^*5*^*,N*^*10*^-tri-feruloyl-spermidine, (**d**) *N*^1^,*N*^5^,*N*^10^-tri-(hydroxyferuloyl)-spermidine, (**e**) *N*^1^,*N*^5^-di-(hydroxyferuloyl)-*N*^10^-sinapoyl-spermidine. Results are the mean±s.e. of three independent determinations. Reversible activity of the spermidine:hydroxycinnamoyl transferase acting upstream of CYP98A9 presumably accounts for the lower accumulation of the *N*^*1*^*,N*^*5*^*,N*^*10*^-tri-coumaroyl-spermidine in the *cyp98a9* mutant compared with the accumulation of *N*^*1*^*,N*^*5*^*,N*^*10*^-tri-feruloyl-spermidine in *cyp98a8*.

To address this question we took advantage of a recently released *A. thaliana cyp98a9* insertion mutant (Supplementary Fig. 4) and compared its young flower bud phenolic profile to those of the previously characterized *cyp98a8* insertion line and wild-type plants. Ultra-performance liquid chromatography-tandem mass spectrometry (UPLC-MS/MS) profiles of the flower buds revealed a dramatic decrease in the major phenolamide compound *N*^*1*^*,N*^*5*^-di-(hydroxyferuloyl)-*N*^*10*^-sinapoyl-spermidine in both the *cyp98a8* and *cyp98a9* single mutants, with a concomitant accumulation of *N*^*1*^*,N*^*5*^*,N*^*10*^-tri-feruloyl-spermidine in *cyp98a8.* The latter compound was completely absent in cyp*98a9* buds (Fig. 2). This confirmed that subfunctionalization of CYP98A8 and CYP98A9 in the phenolamide pathway occurred in lineage I. The complete partitioning of functions *in vivo,* when 3′-hydroxylase activity is shared by both enzymes tested *in vitro,* implied a modification in protein–protein interaction resulting in spatial reorganization of partner enzymes in the tapetal cells. We then hypothesized that the split of function we detected in lineage I most likely contributed to the retention of *CYP98A9*.

### Molecular evolution of the *CYP98* duplicates in Brassicales

To gain some insight into the molecular evolutionary mechanism that led to functional shifts in *CYP98A8* and *CYP98A9*, we compared rates of non-synonymous to synonymous codon substitutions (omega, *ω*=*d*_N_/*d*_S_) along several branches of the Brassicales *CYP98* phylogeny. Both the branch model and the clade model of Codeml analyses indicated that all *CYP98* sequences were under purifying selection (all *ω*<0.4, and even 0.2 for the branch model) and that the lineage I *CYP98A8* sequences were significantly more constrained than those of *CYP98A9* and *CYP98A8* from lineages II and II (Fig. 1a; Supplementary Note 1; Supplementary Table 2). Interestingly, when applying the free-ratio model of Codeml (independent *ω* ratio for each branch), we detected a relaxation of selection occurring along the branch leading to the *CYP98A8* clade (*ω*=0.63; Supplementary Fig. 5), which could reflect the acquisition of the 5′-hydroxylase activity in CYP98A8. With a single exception, *ω* values were below 1; *ω* for lineage I *CYP98A3* and *CYP98A8* sequences was lower than for *CYP98A8s* from lineages II and III. The highest *ω* was associated to the basal internal branch of the *CYP98A9* clade that showed evidence of relaxed selection (*ω*=1.01; Supplementary Fig. 5), which suggested the possibility of a change of function emerging post-duplication in *CYP98A9s*. A McDonald–Kreitman test comparing within-population polymorphism with between-species divergence^26^ for *A. thaliana* and *L. alabamica CYP98A9s* showed a significant signature of purifying selection acting on *A. thaliana* CYP98A9 proteins (Supplementary Table 3). All models, including Codeml branch-site and Fitmodel analysis (Supplementary Note 1; Supplementary Table 2), failed to detect a significant signature of positive selection along any branch of the tree and/or sequence sites.

### *CYP98A8* and *CYP98A9* wire to different regulatory networks

**Figure 3:**
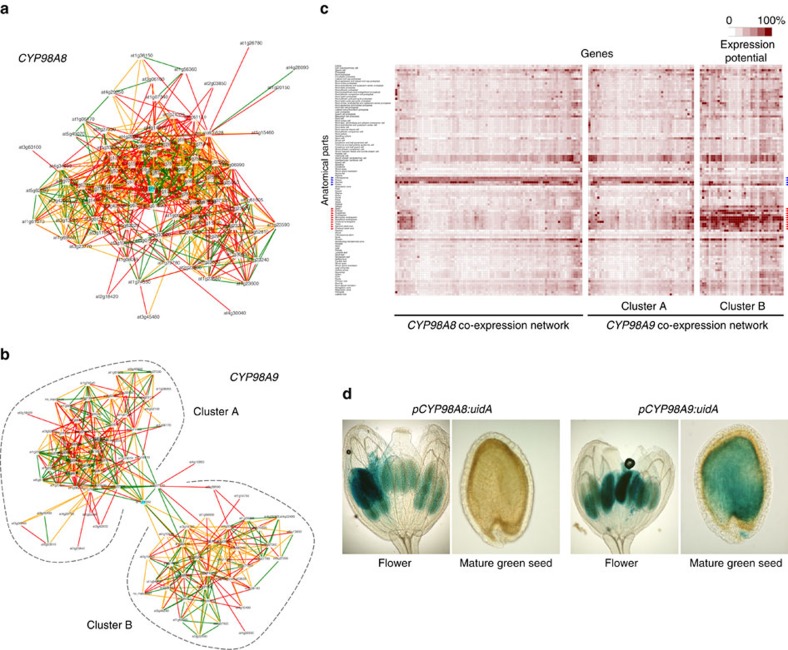
Expansion of *CYP98A9* expression network in *A. thaliana*. (**a**) *AtCYP98A8* and (**b**) *AtCYP98A9* co-expression networks. *AtCYP98A9* is associated with two distinct co-expression clusters, labelled A and B. (**c**) Hierarchical clustering of genes involved in the different co-expression networks. Each element of the matrix represents the expression potential of a given gene (column) in a defined anatomic compartment (line). Blue arrowheads indicate flower- and male reproductive tissue-related samples. Red arrowheads indicate seed-related samples. (**d**) Analysis of *pCYP98A8:uidA* and *pCYP98A9:uidA* expression patterns in opening flowers and green seeds.

To examine the possibility of a change of function emerging in *CYP98A9* after duplication, we first analysed in more detail the *CYP98A9* expression pattern in *A. thaliana. CYP98A9* was previously shown to share *CYP98A8* expression during pollen development, but its promoter was also active in root tips and revealed weak expression in vascular tissues^18^. Functional divergence following the tandem duplication is further suggested by a co-expression analysis indicating a strong correlation of *CYP98A8* expression with a major network of genes involved in flower development, whereas *CYP98A9* expands to an additional network related to seeds (Fig. 3a–c; Supplementary Table 4). This observation was confirmed by β-glucuronidase (GUS) staining, which showed *CYP98A9* promoter activity in mature, green seed endosperm (Fig. 3d), consistent with a recent microarray analysis^27^. In sum, both our own and published expression data indicate that the role of *CYP98A9* reached beyond pollen metabolism.

### CYP98A9 gained an additional activity in flavonoid metabolism

**Figure 4:**
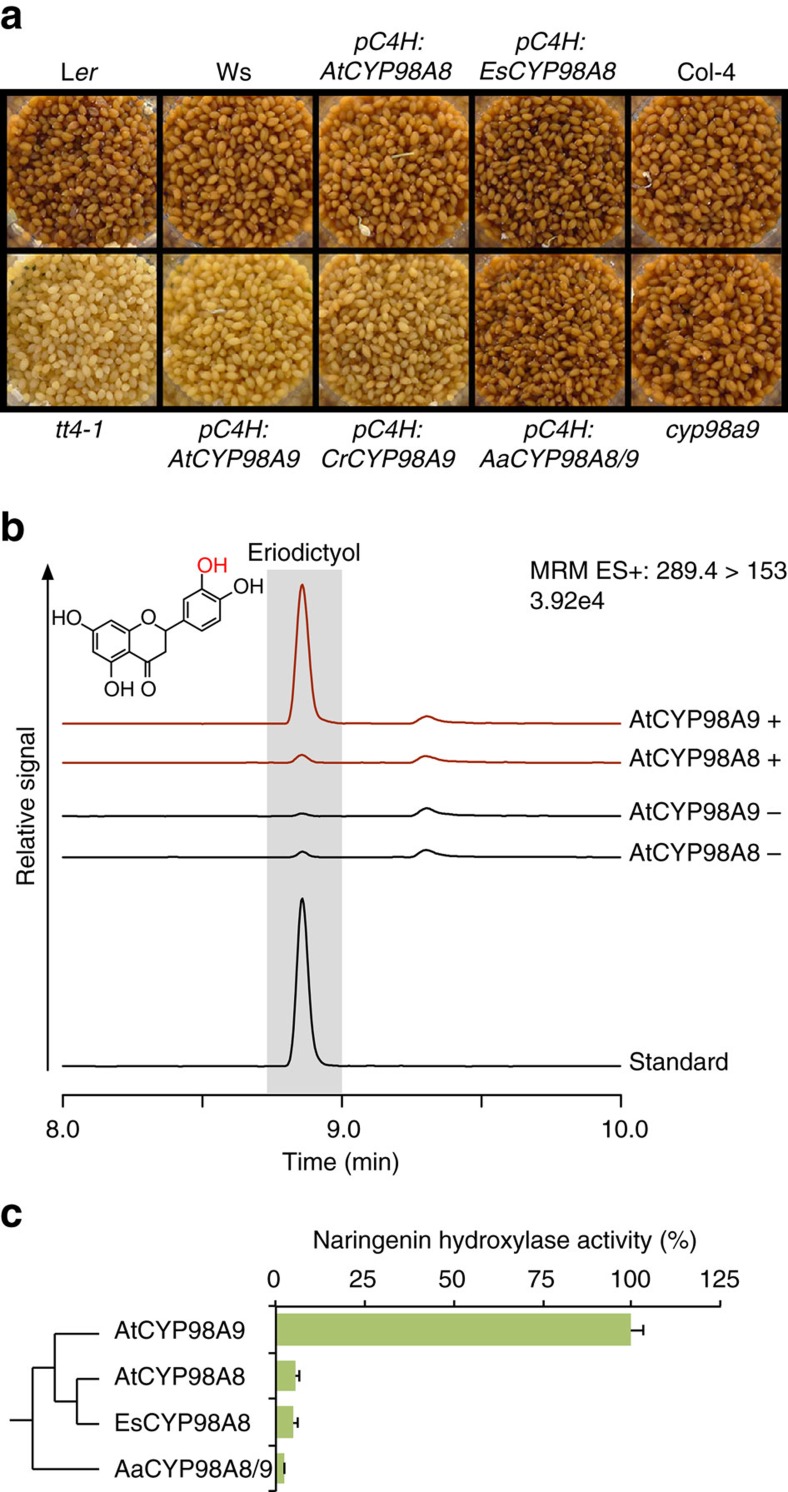
CYP98A9 acquired more pleiotropic activity favouring flavonoid metabolism. (**a**) Colour of the seeds from *A. thaliana* transgenic lines expressing *CYP98A8/A9* genes under control of the *A. thaliana C4H* promoter. Only expression of lineage I *CYP98A9* leads to a *transparent testa* (*tt*) phenotype. Col-4: wild-type Arabidopsis Columbia-4. L*er*: wild-type Arabidopsis Landsberg *erecta*. Ws: wild-type Arabidopsis Wassilewskija. *tt4-1*: *transparent testa 4* mutant for the chalcone synthase gene. (**b**) *In vitro* enzyme assays showing the CYP98A9-dependent conversion of naringenin into eriodictyol. Assays with (+) and controls without (−) NADPH were performed. Eriodictyol (grayed elution time) was detected using the *m/z* 289.4>153.0 transition in multiple reactions monitoring (MRM) positive mode. (**c**) Relative naringenin hydroxylase activity (% relative to highest activity set to 100). Error bars represent the s.e. from three independent assays. Aa, *Aethionema arabicum*; At, *Arabidospsis thaliana*; Es, *Eutrema salsugineum*.

To reveal potential additional functions and overlooked activities of CYP98A9, we used *CYP98A9* loss and gain of function lines. The latter were generated using the promoter of the upstream P450 gene in the phenolic pathway, encoding the cinnamic acid 4-hydroxylase (*C4H*)^28^ that provides precursors for the lignin, phenolamide and flavonoid pathways. A similar *CYP98A8* overexpression construct was used as control. We observed no differences in development, seed set or colour, and fertility between wild-type and *cyp98a9* null mutants grown under controlled conditions. No obvious alteration in plant growth, seed set and phenotype was likewise detected for *pC4H:AtCYP98A8*. Conversely, seeds from *pC4H:AtCYP98A9-*transformed plants showed a typical *transparent testa (tt)* phenotype (yellow instead of brown seeds) (Fig. 4a), typical of seed coats deficient in flavonoids and tannins^29^,^30^. Vanillin seed staining of epicatechins and proanthocyanidins^31^ confirmed that only *CYP98A9* expression led to a seed coat tannins defect (Supplementary Fig. 6a). Moreover, UPLC-MS/MS profiling of soluble flavonoids indicated that *AtCYP98A9* expression resulted in a complete depletion in quercetin-3-*O*-rhamnoside (Supplementary Table 5), the major soluble seed flavonol in *A. thaliana*^32^. *CYP98A9* expression thus impacts soluble flavonoid metabolism and not just condensed tannin composition or polymerization. However, no significant alteration in whole seed, flower buds or developing siliques flavonoids was detected in the insertion mutant *cyp98a9* (Fig. 4a; Supplementary Figs 6b and 7), but time- or cell-specific alteration in flavonoid content in this mutant cannot be excluded.

**Figure 5:**
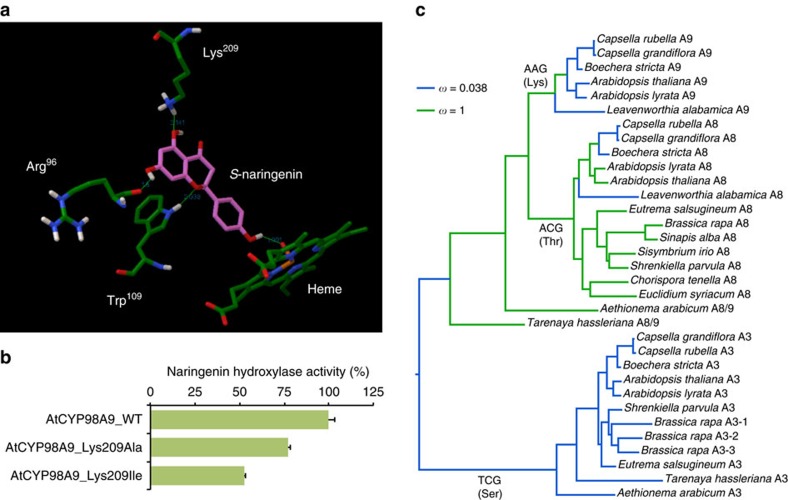
Lys^209^ is a determinant for CYP98A9 flavonoid hydroxylase activity. (**a**) Docking of *S*-naringenin in the AtCYP98A9 active site. Arg^96^, Trp^109^ and Lys^209^ anchor naringenin for B-ring 3′-hydroxylation. (**b**) Lys^209^ substitution with alanine or isoleucine reduces AtCYP98A9 naringenin hydroxylase activity *in vitro*. Error bars represent the s.e. from three independent assays. (**c**) Fitmodel reconstitution of the history of the selection pressure on the *AtCYP98A9* Lys^209^ codon. The most frequent codon and amino acid are indicated on branches.

To determine if this flavonoid-impacting function was specific to *A. thaliana CYP98A9*, overexpression constructs of another lineage I *CYP98A9* (*CrCYP98A9*, *Capsella rubella),* and of representative members of lineage II *CYP98A8* (*EsCYP98A8, E. salsugineum*) and of the ancestral *AaCYP98A8/9* (*A. arabicum*) were tested for their impact on seeds. Only expression of *C. rubella CYP98A9* prevented seed flavonoid accumulation (Fig. 4a; Supplementary Fig. 6b). The capacity to impair flavonoid accumulation was thus likely acquired by *CYP98A9* before the radiation of lineage I. We then set out to identify the flavonoid substrate of CYP98A9s likely to explain the *transparent testa* phenotype. *In vitro* enzymatic screens of flavonoid candidates spotted the AtCYP98A9-dependent conversion of naringenin into eriodictyol (that is, 3′-hydroxy-naringenin), a reaction very poorly catalysed by AtCYP98A8, EsCYP98A8 and AaCYP98A8/9 (Fig. 4b,c). AtCYP98A8 and AtCYP98A9 homology modelling and docking experiments in addition confirmed that only AtCYP98A9 efficiently anchored *S*-naringenin in its active site, with B-ring oriented for 3′-hydroxylation (Fig. 5a; Supplementary Fig. 8). From the three residues critical for naringenin stabilization, only Lys^209^ was found discriminant throughout the CYP98A8s/CYP98A9s alignment (Supplementary Fig. 9). The involvement of Lys^209^ in AtCYP98A9 naringenin hydroxylase activity was supported by Lys^209^ substitution with uncharged amino acids (Ala and Ile) leading to up to 50% selective decrease in flavonoid hydroxylase activity (Fig. 5b). According to Fitmodel results (MX+S1 model, Fig. 5c; Supplementary Note 1; Supplementary Table 2) used to estimate different *d*_N_/*d*_S_ ratios to each sequence site and branch on the phylogenetic tree (see Methods), this site was constrained in CYP98A9 sequences (*ω*=0.038—codon AAG in the six species analysed; Supplementary Fig. 9), but evolved neutrally (*ω*=1.000) at the basal branch of the A9 clade and in most of the other branches of the A8A9 clade (Fig. 5c). Lys^209^ and the surrounding residues that also vary between CYP98A8 and CYP98A9 might thus be instrumental in the further evolution of CYP98A9 function.

It is possible that the novel CYP98A9 function is still latent, yet flavonoids are regarded as important determinants of plant fitness as ultraviolet protectors, antimicrobials and antifeedants^33^. They are reported to impact seed germination and growth^34^. It thus can also be postulated that the new function of CYP98A9 contributed to its retention in lineage I.

## Discussion

**Figure 6:**
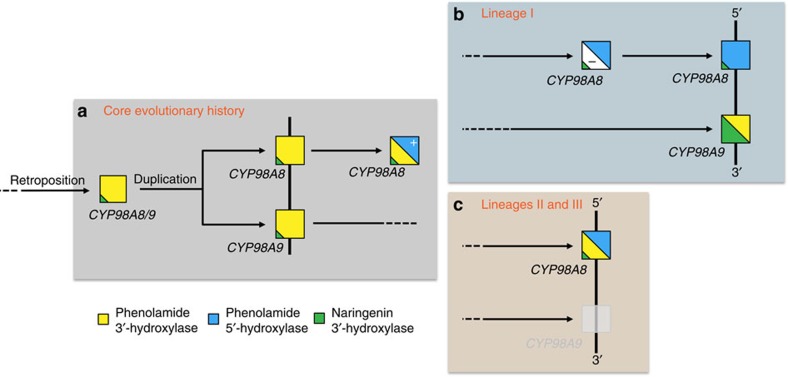
Model of the *CYP98A8/CYP98A9* evolution in Brassicaceae. (**a**) Initial steps of the *CYP98A8/9* evolution shared by lineages I, II and III. The initial CYP98A3 retroposition event in Brassicales (100-70 Ma) led to the *CYP98A8/9* ancestral gene, which became a phenolamide 3′-hydroxylase. The retroposition was followed by a tandem gene duplication in Brassicaceae around 45 Ma leading to the *CYP98A8* and *CYP98A9* sister genes. CYP98A8 then acquired an additional function, the phenolamide 5′-hydrolase activity. (**b**) Further evolution of *CYP98A8* and *CYP98A9* in the lineage I of the Brassicaceae, CYP98A8 lost the phenolamide 3′-hydroxylase activity *in vivo*, and maintained only the 5′-hydroxylase activity. Meanwhile, CYP98A9 maintained the 3′-hydroxylase activity, and simultaneously gained naringenin 3′-hydroxylase activity. (**c**) Further evolution of *CYP98A8* and *CYP98A9* in the lineages II and III of the Brassicaceae, in lineages II and III, the *CYP98A9* copy was lost, and CYP98A8 maintained both 3′- and 5′-hydroxylase activities.

In summary, our work provides an unprecedented example of multistep and divergent evolution of a retrogene expressed in male reproductive tissues, where successive duplication, along with acquisition and partitioning of a dual function, led to progressive, lineage-specific expansion of phenolic metabolism, and emergence of new and diverse metabolic capacities in the plant. Our experimental data are fully supported by molecular evolutionary results and highlight the rise of an extended gene activity simultaneously rewiring gene expression networks and leading to an enzyme with pleiotropic activity. Metabolic profiling of plant tissues and ectopic gene expression provide a practical demonstration that *in vitro* enzyme activity and gene expression in a single context are not sufficient to describe gene function. Our data, in addition, reveal a striking case of divergent evolution of paralogs in sister lineages (Fig. 6), illustrating how relaxation of purifying selection following gene duplication can allow for the exploration of novel metabolic pathways.

## Methods

### Plant materials and growth conditions

Unless otherwise stated, plants were grown on soil under a 16h/8h (day/night) regime with 70% humidity. The *cyp98a8* insertion mutant (SALK_131366) was isolated and characterized previously^18^. The *cyp98a9* insertion mutant (#SK27279) was obtained from the Saskatoon transfer DNA collection^35^. The correct transfer DNA insertion in the *CYP98A9* gene was confirmed by PCR-based genotyping and further validated by the drastic decrease in *CYP98A9* expression observed in the mutant by quantitative reverse transcription PCR (qRT–PCR) (Supplementary Fig. 4). Primers used for the mutant validation are listed in Supplementary Table 6. For parent ion scan analysis, flower buds from *A. thaliana* and *E. salsugineum* grown under controlled conditions were harvested. Flower buds from *T. hassleriana* were a kind gift from Pr. Eric Schranz (Wageningen University). *Bunias orientalis* and *A. grandiflorum* were sampled in the botanical garden of the University of Strasbourg.

### Cloning procedures and production of transgenic lines

The intronless coding sequences of *AtCYP98A8*, *AtCYP98A9*, *CrCYP98A9, EsCYP98A8* and *AaCYP98A8/9* were PCR-amplified from genomic DNA using Gateway compatible primers and introduced into pDONR207 vector by BP reaction to generate pENTRY clones. Expression constructs *pC4H:AtCYP98A8*, *pC4H:AtCYP98A9, pC4H:CrCYP98A9, pC4H:EsCYP98A8* and *pC4H:AaCYP98A8/9* were obtained by recombining the corresponding pENTRY with the pCC0996 Gateway destination vector that contains a 3 kb promoter fragment of *A. thaliana Cinnamate 4-Hydroxylase* (*pC4H*)^36^ using LR clonase (Invitrogen). Primers used for molecular cloning are listed in Supplementary Table 6. *Agrobacterium tumefaciens* GV3101 carrying the expression vector was used to transform *Arabidopsis* wild-type plants (Ws background) by dipping immature inflorescences in a bacterial suspension^37^. Transgenic plants were selected on soil via Basta treatment (250 mg l^−1^; Agro Evo). T2 seeds were used for subsequent analyses.

### Amplification of CYP98A region from lineage III species

To obtain *CYP98A8* and *CYP98A9* sequence data from species in Brassicaceae lineage III, we performed multiple rounds of high-efficiency thermal asymmetric interlaced PCR (hiTAIL-PCR)^38^. Initial nested primer sets were designed to anneal to regions highly conserved in known Brassicaceae *CYP98A8* and *CYP98A9* sequences. Polymorphic sites were incorporated into primers, and thus the resulting primers were degenerate at several nucleotide positions. Amplicons were sequenced and used to inform sequence-specific primer design to walk upstream and downstream along the chromosome using additional rounds of hiTAIL-pPCR^38^. Primers are available in Supplementary Table 6.

*E. syriacum* and *C. tenella* DNA samples were those used in Beilstein *et al.*^39^. Nested amplification was performed in a two-step process according to the hiTAIL-PCR protocol^38^. The process was repeated until the assembled reads reached flanking genes. The software CoGe^40^ was used to compare assembled sequence reads for *E. syriacum* and *C. tenella* with published whole-genome sequences from Brassicaceae lineage I and II species.

PCR products were separated on a 1% agarose gel with low EDTA before excision, filtration (Millapore Centrifugal Filter Units), and ethanol precipitation. Purified PCR products were ligated into pGEM-T Easy vector (Promega) and transformed into TOP10 competent cells and plated on selective media for screening. Positive transformants were cultured overnight in liquid media, and plasmid DNA harvested using an alkaline lysis kit (Zymo Research). Cloned fragments were sequenced in both directions to achieve a minimum of 85% overlap. Overlapping sequence reads were assembled into a consensus sequence using the software Geneious v. 6.6 (Biomatters).

### Recombinant CYP98 protein production in yeast

Intronless coding sequences of *EsCYP98A8, AaCYP98A8/9* and *ThCYP98A8/9* genes were PCR-amplified from genomic DNA using USER-compatible primers^41^. Amplicons were subsequently introduced in the pYeDP60u2 vector^42^ and verified by sequencing. For *AtCYP98A8* and *AtCYP98A9* pYeDP60 plasmids construction, the corresponding coding sequences were PCR-amplified from genomic DNA and introduced between the BamHI and KpnI sites and BamHI and EcoRI sites, respectively, as described previously^18^. Primers used for molecular cloning are visible in Supplementary Table 6.

The *Saccharomyces cerevisiae* WAT11 strain, expressing the *ATR1* cytochrome P450 reductase from *A. thaliana*^43^, was transformed with pYeDP60 vectors and selected on minimum SGI medium (1 g l^−1^ bactocasamino acids, 7 g l^−1^ yeast nitrogen base, 20 g l^−1^ glucose and 40 mg l^−1^ L-tryptophan). Liquid cultures were initiated from selected colonies grown on SGI for 18 h at 28 °C under agitation (160 r.p.m.). Ten milliliters of SGI culture were used to inoculate 200 ml of YPGE (10 g l^−1^ bactopeptone, 10 g l^−1^ yeast extract, 5 g l^−1^ glucose, 3% ethanol by volume). After 30 h growth at 28 °C at 160 r.p.m., recombinant proteins production was induced by supplementing the medium with 10 ml of 200 g l^−1^ galactose and further incubation at 20 °C for 16 h.

Yeast cells were harvested by centrifugation at 7,500*g* for 10 min at 4 °C, washed with TEK buffer (50 mM Tris-HCl pH 7.5, 1 mM EDTA, 100 mM KCl) and resuspended in 2 ml of TES (50 mM Tris-HCl pH 7.5, 1 mM EDTA, 600 mM sorbitol) supplemented with 5 mM 2-mercaptoethanol and 10 g l^−1^ bovine serum albumin, fraction V (buffer A). Cell suspensions were transferred to 50 ml conical tubes and homogenized with 0.5 mm glass beads by handshaking five times for 1 min each. Beads were washed twice with 30 ml of cold buffer A. Cell debris and remaining glass beads were removed from the pooled lysates by 20 min centrifugation at 7,500*g* and 4 °C. Supernatant was filtrated on Miracloth (22–25 μm pore size, Calbiochem, Maryland) and microsomal fraction pelleted by centrifugation at 100,000*g* and 4 °C for 1 h. Pelleted membranes were resuspended in TEG buffer (50 mM Tris-HCl pH 7.5, 0.5 mM EDTA, 30% glycerol by volume) with a Potter-Elvehjem homogenizer. Microsomal membranes were stored at −20 °C until processing.

### Enzyme assays

Enzyme assays were conducted in 100 μl final volume, containing 20 mM potassium phosphate buffer (pH 7.4), 200 μg microsomal proteins, 100 μM substrate and 500 μM NADPH. Reactions were initiated with NADPH addition, incubated for 30 min at 28 °C in the dark and terminated with 10 μl of 50% acetic acid. Samples were centrifuged at 13,000*g* for 5 min to pellet microsomal membranes and supernatants were used for product analysis. Hydroxycinnamoyl-spermidines used as substrates and chromatographic references were chemically synthesized^18^. For naringenin 3′-hydroxylation, activity was normalized to the activity recorded for the same enzyme with tri-*p*-coumaroyl-spermidine and expressed as a percentage of the maximal activity.

### Extraction and analysis of phenolamides and flavonoids

For flavonoid analysis, mature dry seeds were ground in a 2 ml safe-locker tube with 1 ml of 75% acetonitrile for 5 min using a TissueLyser II (Qiagen) at 4 °C. The extracts were sonicated for 20 min on ice. After centrifugation (14,000*g*, 15 min), the pellet was further extracted with 1 ml of 75% acetonitrile by overnight shaking at 4 °C. The two extracts were pooled, dried under vacuum and resuspended in 200 μl of 75% acetonitrile before UPLC-MS/MS analysis^44^.

To release flavonoid aglycones, crude extracts were dried *in vacuo*. Dry residues were re-suspended in a 50% methanol solution containing 2 N HCl and 2.5 mg ml^−1^ ascorbic acid as an antioxidant. Acid hydrolysis was performed at 80 °C for 2 h. Samples were cooled down before UPLC-MS/MS analysis.

For the analysis of phenolamides, samples were first extracted with 1 ml of ice-cold 80% methanol. After centrifugation, the supernatant was recovered and the pellet further extracted with 1 ml of ice-cold 80% methanol overnight at 4 °C. Extracts were pooled, dried under vacuum and resuspended in 200 μl of 80% methanol before UPLC-MS/MS analysis. The mobile phase consisted of water (A) and acetonitrile (B), both containing 0.1% formic acid. Run started with 1 min of 95% A, followed by linear gradients to reach 53% B at 7 min and 100% B at 8 min, then isocratic conditions using B for 2.5 min. Return to initial conditions was achieved in 3.5 min, with a total run time of 15 min. The column was maintained at 28 °C with a flow rate of 0.4 ml min^−1^, injecting 3 or 5 μl sample. Ultraviolet absorbance was recorded from 200 to 600 nm. MS scans in positive and negative mode were performed, and multiple reactions monitoring were developed for every detected peak. Compounds were identified by comparison of two orthogonal data (RT and MS/MS) relative to authentic compounds analysed under identical experimental conditions, or putatively annotated by comparison of MS/MS spectra with published data^45^,^46^.

### Vanillin assay

Mature dry seeds were incubated in a solution of 1% (w/v) vanillin (Fluka #350346) prepared in 6 N HCl at room temperature for 1 h. Vanillin turns red upon binding to flavan-3,4-diols (leucoanthocyanidins) and flavan-4-ols (catechins), which are present either as monomers or as terminal subunits of proanthocyanidins^31^.

### Determination of gene expression divergence

*pCYP98A8:uidA* and *pCYP98A9:uidA* plants^18^ were incubated in 90% acetone for 20 min on ice, rinsed with water, and transferred to a GUS solution containing 1 mM 5-bromo-4-chloro-3-indolyl-β-D-glucuronide (X-Gluc), 100 mM sodium phosphate (pH 7.0), 10 mM EDTA, 0.5 mM potassium ferricyanide, 0.5 mM potassium ferrocyanide and 0.1% (v/v) Triton X-100. Samples were incubated at 37 °C in the dark overnight. Tissues were cleared three times in 75% ethanol before imaging with a Nikon (ECLIPSE, E800) microscope. For GUS staining of seeds, additional clearing steps were added. Opened siliques were first treated with ethanol:acetic acid (v/v) for 4 h, and then restored in Hoyer's medium before microsopy analysis^47^.

Gene co-expression network analysis was performed with the PlaNet on line tool (http://aranet.mpimp-golm.mpg.de/) using *At1g74540* (*CYP98A8*) and *At1g74550* (*CYP98A9*) as gene id inputs. The gene list corresponding to the different co-expression networks (see Supplementary Table 4) were then subjected to hierarchical clustering based on Pearson correlation coefficient and with optimal leaf-ordering using the Genevestigator embedded tool^48^.

### RNA extraction and gene expression analysis by qRT–PCR

Samples were harvested and snap-frozen in liquid nitrogen. Total RNA was isolated from pooled tissues using NucleoSpin RNA Plant kit (Macherey-Nagel) and subsequently treated with DNase I (Fermentas). In all, 1.5 μg of total RNA was reverse-transcribed with SuperScript III Reverse Transcriptase (Invitrogen) in the presence of 200 ng random primers. qRT–PCR plates were prepared with a Biomek 3,000 pipetting system (Beckman Coulter, Villepinte, France) and run on a LightCycler 480 II device (Roche). Each reaction was prepared using 2 μl of tenfold diluted complementary DNA, 5 μl of LightCycler 480 SYBR Green I Master (Roche) and 250 nM of forward and reverse primers in a total volume of 10 μl. The amplification program was 95 °C for 10 min and 40 cycles (95 °C denaturation for 10 s, annealing at 60 °C for 15 s, extension at 72  °C for 15 s), followed by a melting curve analysis from 55 to 95 °C to check for transcripts specificity. All reactions were performed in triplicate. The *SAND* gene expression was stable among flower samples and thus chosen as internal standard for normalization. Primers used for qRT–PCR detection were previously described^18^ and their sequences are provided in Supplementary Table 6.

### Phylogenetic *d*_N_/*d*_S_ analysis

Coding sequences were aligned with MUSCLE^49^ and phylogenies were built using PhyML^50^ using the generalized time-reversible (GTR) model with gamma distribution (four rate classes of sites with optimized alpha) *via* the Seaview software (http://pbil.univ-lyon1.fr/)^51^. PAML (codeml) (http://abacus.gene.ucl.ac.uk/software/paml.html)^52^ and Fitmodel^53^ were applied to the coding-sequence alignments and phylogenetic trees. All the analyses were run both with a trimmed alignment (404 over 560 codons) where the most variable sites, mainly in the 5′ and 3′ regions of the coding sequences, were suppressed by GBlocks^54^,^55^ and with a longer alignment where all but the gap-containing sites were conserved (457 over 560 codons). We ran ‘Branch model', ‘Site model', ‘Clade model', ‘Branch-site' and ‘Free-ratio' analyses following codeml standard procedures. We compared nested models using the likelihood ratio test framework to test statistical differences in *d*_N_/*d*_S_. We ran Fitmodel M2a+S1 (with *ω*1<*ω*2<*ω*3 and switch between selection regimes allowed) and MX+S1 (with *ω*0≤1; 1<*ω*1≤1.5; *ω*2>1.5 and switch between selection regimes allowed) as an alternative ‘Branch-site' analysis and M2a (no switch between selection regimes allowed) as a null model to perform a likelihood ratio test^53^. All results and associated statistical tests are available in Supplementary Note 1 and Supplementary Table 2.

### Homology modelling

All Modeller and Autodock calculations were performed using the computing facilities of the CEA-DSV (cluster Gabriel) at Saclay.

Three-dimensional models of *A. thaliana* CYP98A8/9 isoforms deprived of their membrane spanning domain (residues 24 to 491 for 98A8, and residues 24 to 485 for 98A9) were built using Modeller9v12 (refs 56, 57) and the crystal structures of CYP2C9 (PDB 1OG2), CYP2A6 (PDB 2PG6), CYP2D6 (PDB 2F9Q), CYP2B4 (PDB 3MVR), and CYP2C5 (PDB 1DT6) as templates. The selection of the template structures for CYP98A8/9 model rebuilding followed four criteria: the best similarity according to PDB-Blast, the highest alignment length, the atomic resolution and ligand-free structures for getting undeformed states. These mammalian CYPs exhibited rather low sequence identities with AtCYP98A8 isoforms (24 to 25%), but the three-dimensional structures are well-aligned by MUSTANG algorithm^58^, and the resulting alignment was consistent with that obtained with MAFFT-L-INS-I algorithm^59^. The validated sequence alignment used as input for Modeller is available in Supplementary Fig. 10.

For each isoform, two runs of 100 models were performed with a further loop refinement protocol, and the generated models sorted by the MODELLER objective function were evaluated by their discrete optimized protein energy and GA341 scores calculated by Modeller. For each isoform, the five best models (corresponding to the lowest discrete optimized protein energy scores) issued from each run were pooled and submitted to the online metaserver SAVES (structural analysis and verification server (http://services.mbi.ucla.edu/SAVES), and finally to the QMEAN scoring function^60^ server^61^ for model quality assessment. The final model was the best one according to a good compromise between the scores calculated by the SAVES server scoring programs and the QMEAN value. As a result, the selected AtCYP98A8 and AtCYP98A9 models had a QMEAN score equal to 0.701 and 0.689, respectively, which are good scores when compared to the QMEAN scores of individual PDB templates.

### Docking experiments

The protein structures generated by Modeller were first stripped of all hydrogen atoms, and then atom charges and hydrogen atoms were added to the protein with the UCSF Chimera package (www.cgl.ucsf.edu/chimera)^62^ using AMBER ff99SB parameters. The first parameters applied for the heme (defined as Fe^III^ protoporphyrin IX) were obtained from Oda *et al.*^63^, and in the docking experiments with S-Naringenin (Nar) the heme cofactor was defined as the highly reactive intermediate *compound I* (Fe^IV^=O)^+^ by using the parameters (geometry and atom charges) of an AMBER-compatible heme model developed by Shahrokh *et al.*^64^. The atom charges of the proximal thiolate were taken from the same work.

Molecular docking experiments with the flavonoid *S*-naringenin (Nar) at the active site were performed using AutoDock 4 (release 4.2.6) in the semi-flexible mode, and prepared with AutoDock Tools^65^. The ligand molecule, Nar, was parametrized under Maestro 9.2 (www.schrödinger.com) molecular modelling suite and the structure saved under MOL2 format as input files for AutoDock. Partial charges were assigned using OPLS 2005 force field. There is only one rotatable bond in Nar, but several conformations of the non-aromatic ring have been generated as starting point for docking to allow better conformational sampling under Autodock 4, since the algorithm cannot handle ring flexibility. The MOL2 format files, created in Chimera for the receptor and Maestro for the ligand, were converted into the PDBQT format file by AutoDockTools, which merges all nonpolar hydrogen atoms to the carbon atoms they are bonded to. The receptor was kept rigid.

The docking box, in which grid maps were computed using program AutoGrid^65^, included the active site with the iron-protoporphyrin group on one edge, and the whole distal moiety of the enzyme, including access channels and protein surface, to allow a large sampling of potential poses. The grid built by AutoGrid included 100, 70 and 86 points in x, y and z directions, with a grid spacing of 0.37Å to allow a good compromise between resolution of the explored volume and the size of the binding area. For each Nar conformer, 500 independent runs were performed using the Lamarckian genetic algorithm^66^. The default settings were used for all other parameters.

The poses were assigned a score calculated by Autodock that can be considered as an estimated free energy of ligand binding (indicative of binding affinity), then clustered as a function of the closeness of their positions and conformations with root mean square deviation set at 2.0 Å, and finally ranked by their binding score (for the best pose in the cluster). The resulting histograms of 500 poses of Nar docked into AtCYP98A8 and AtCYP98A9 are visible in the Supplementary Fig. 11. Remarkably, whereas the docking computational conditions were identical for both isoforms, the solutions found for AtCYP98A9 are gathered in the same cluster, within 2 Å of root mean square deviation of clustering, while the histogram of AtCYP98A8 displays a more scattered profile. The best clusters, corresponding to the lowest (that is, the most negative) energetic binding scores, were well-resolved in the histogram of scores for AtCYP98A8 but corresponded to non-productive metabolism positions. All clusters under −4 kcal mol^−1^ were independently investigated for their consistency with productive position for 3′-hydroxylation reaction.

### Data availability

The data that support the findings of this study are available from the corresponding author upon request. *CYP98A8* sequence data from *E. syriacum* and *C. tenella* have been deposited in GenBank with accession codes KX754460 and KX754461.

## Additional information

**How to cite this article**: Liu, Z. *et al.* Evolutionary interplay between sister cytochrome P450 genes shapes plasticity in plant metabolism. *Nat. Commun.* **7**, 13026 doi: 10.1038/ncomms13026 (2016).

## Supplementary Material

Supplementary Information Supplementary Figures 1-11, Supplementary Tables 1-6 and Supplementary Note 1
