# Supplementary material for: Evolutionary interplay between sister cytochrome P450 genes shapes plasticity in plant metabolism
Source: Nat Commun. 2016 Oct 7;7:13026. doi: 10.1038/ncomms13026 (PMC5059761; doi:10.1038/ncomms13026)
Supplement: Supplementary Information — Supplementary Figures 1-11, Supplementary Tables 1-6 and Supplementary Note 1 [file ncomms13026-s1.pdf]

Supplementary Figures

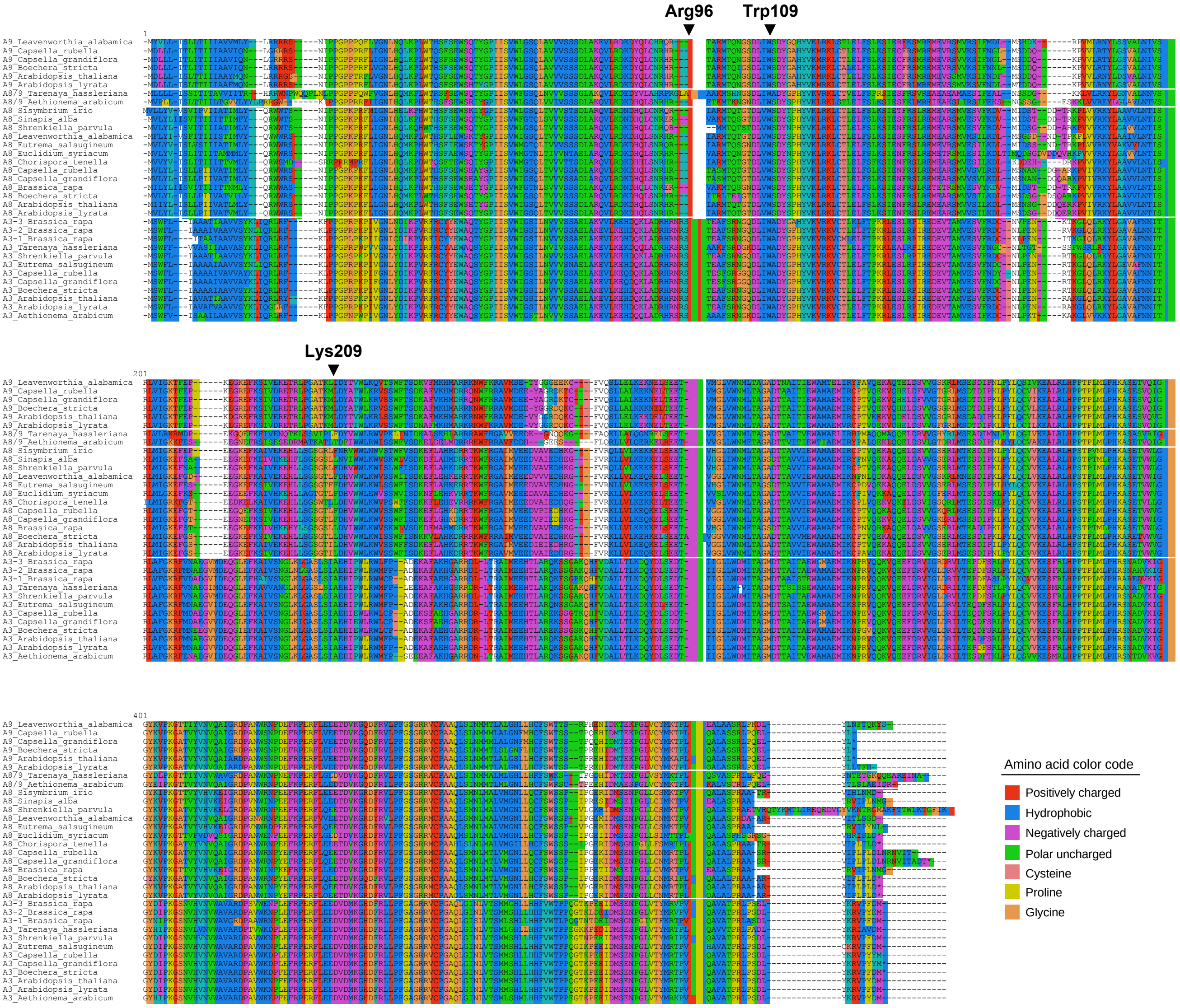

Supplementary Figure 1. ***Brassicales* CYP98 protein sequences alignment.**

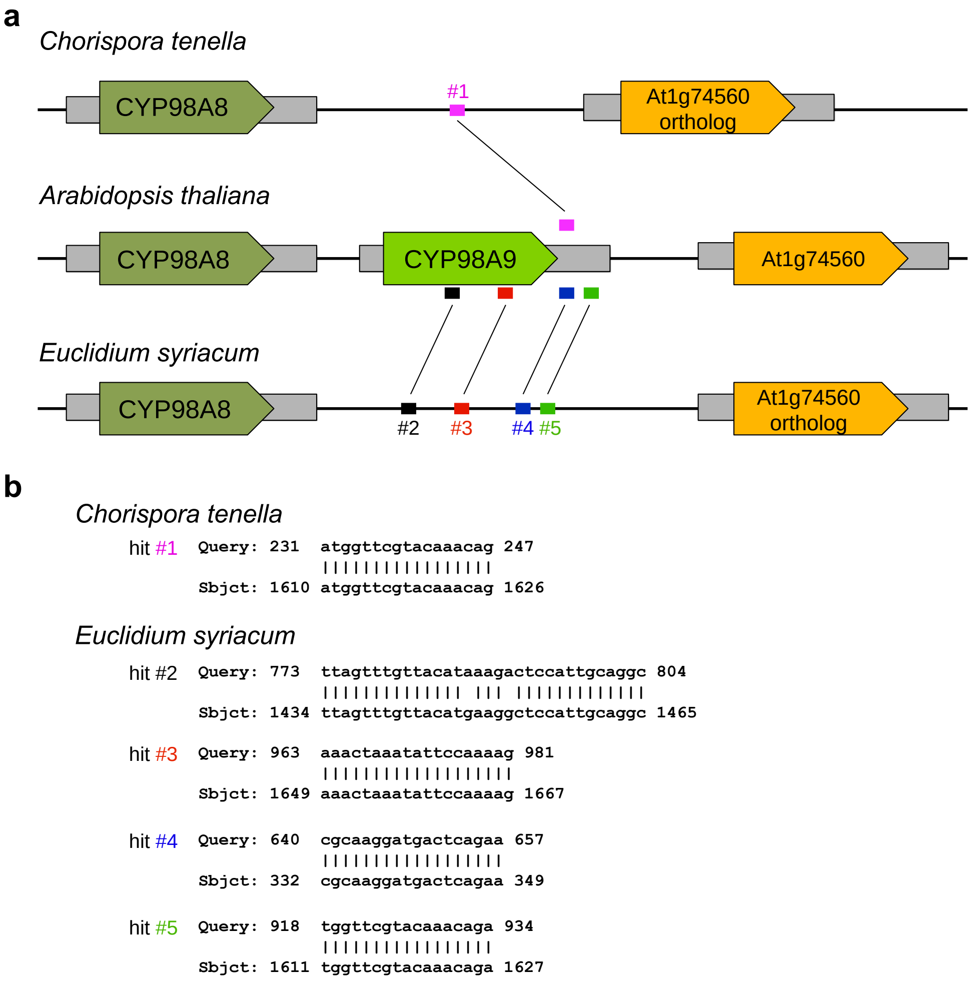

Supplementary Figure 2. Search for *CYP98A8/A9* homologs in the Brassicaceae lineage III species *Chorispora tenella* and *Euclidium syriacum.*

BLAST search respectively found one and four correctly ordered remnant *CYP98A9* fragments in *C. tenella* and *E. syriacum*. (**a**) Schematic view of the *CYP98A8/CYP98A9* genomic region. (**b**) Details of the BLAST hits. BLAST query consisted of *C. tenella* or *E. syriacum* sequences.

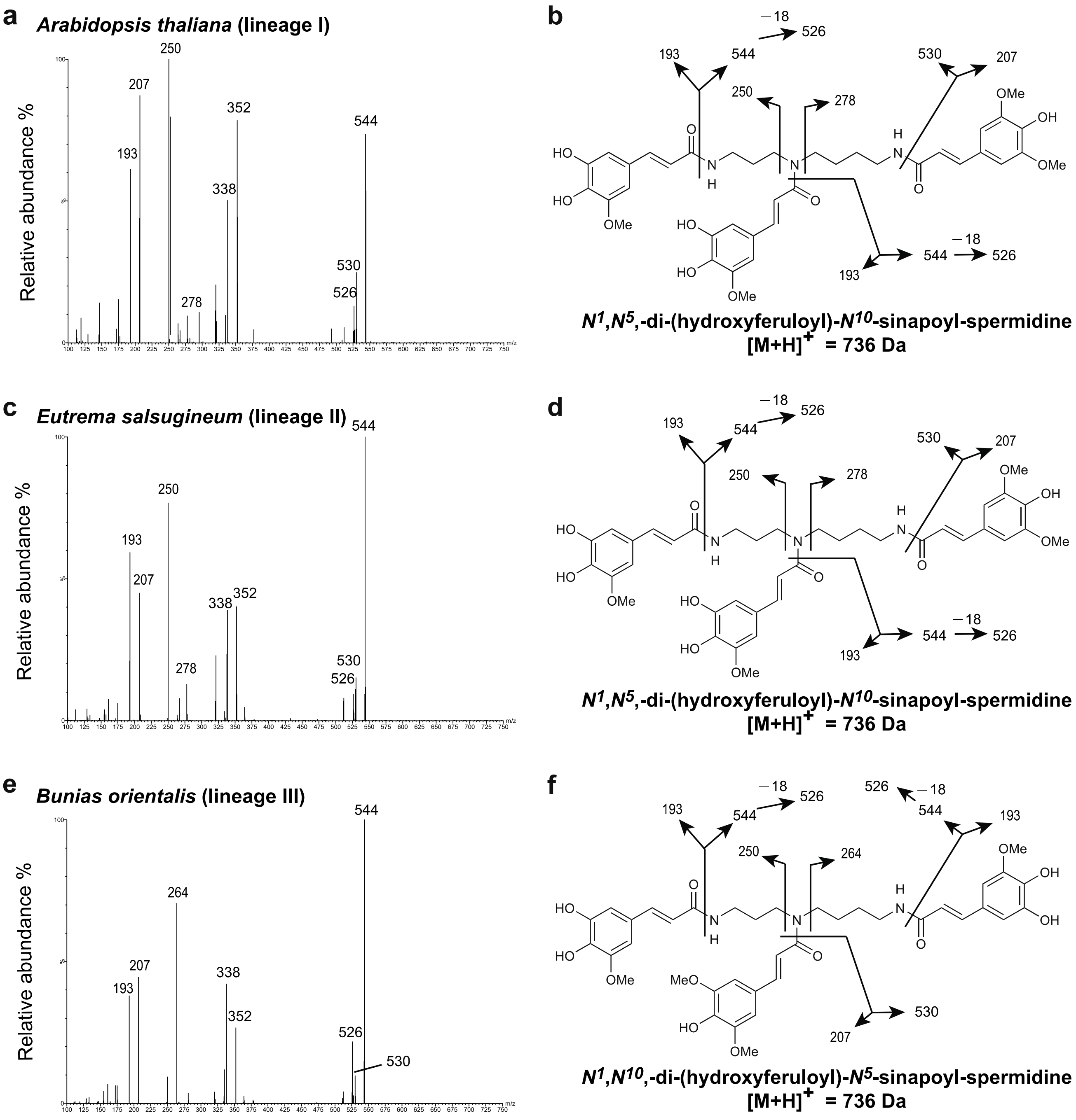

Supplementary Figure 3. Identification of the sinapoylated phenolamide in *Brassicaceae* lineage I, II and III species.

Structure of the molecules (*m/z* 736) in **Fig. 1c** was inferred from their fragmentation patterns obtained by daughter scan analysis of flower bud extract. (**a-b**) *Arabidopsis thaliana* (**a**) fragmentation chromatogram and (**b**) deduced fragmentation pattern. (**c-d**) *Eutrema salsugineum* (**c**) fragmentation chromatogram and (**d**) deduced fragmentation pattern. (**e-f**) *Bunias orientalis* (**e**) fragmentation chromatogram and (**f**) educed fragmentation pattern.

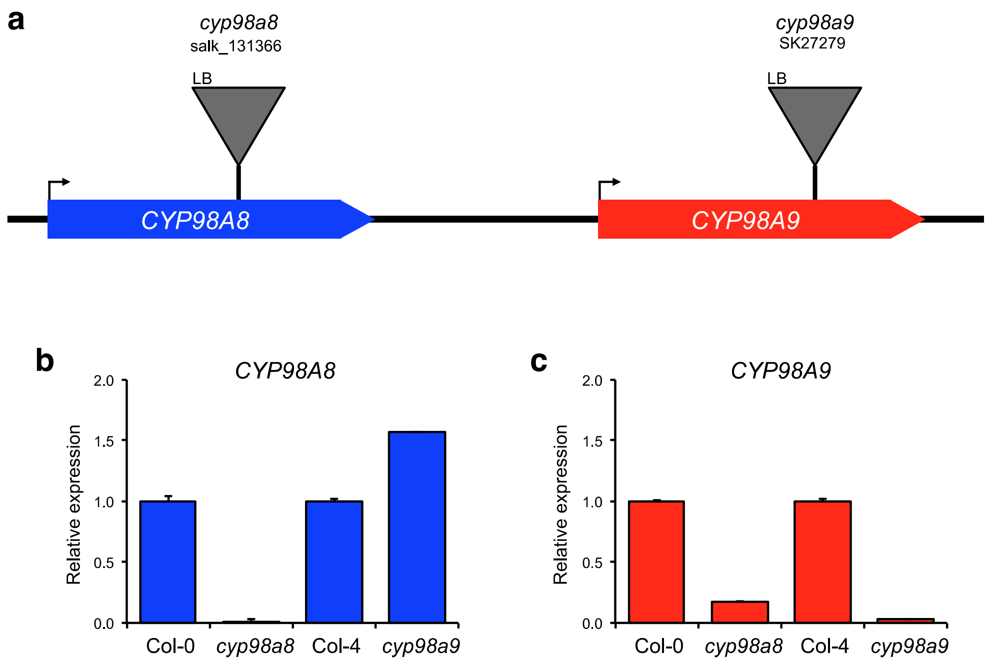

Supplementary Figure 4. Molecular characterization of *Arabidopsis thaliana* insertion mutant lines.

(**a**) Cartoon depicting the *Arabidopsis thaliana CYP98A8*/*CYP98A9* loci organization. Expression level analysis of (**b**) *CYP98A8* (blue) and (**c**) *CYP98A9* (red) confirmed that *cyp98a8* (Col-0 background) and *cyp98a9* (Col-4 background) insertion lines are true KO mutants. Note that a decrease in *CYP98A9* expression was observed in *cyp98a8* mutant, likely as a result of either a detrimental effect of T-DNA insertion or a negative transcriptional feedback. Results are the mean ± SE from three technical replicates.

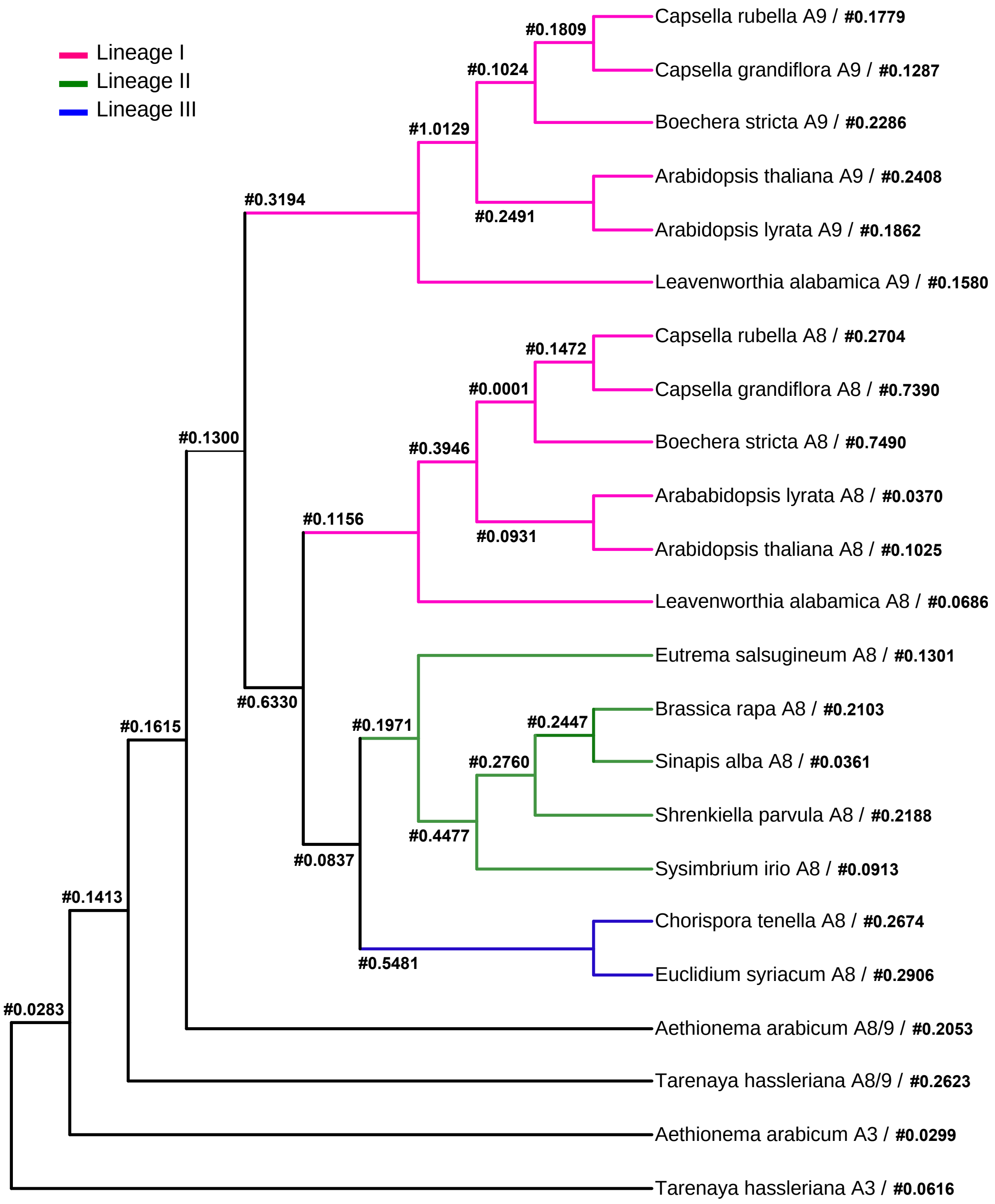

Supplementary Figure 5. Codeml Free Ratio sub-tree.

Omega values are indicated in bold next to branches or next to leaves for terminal branches. lnL0 = -15473.924778; lnL = -13886.83423.

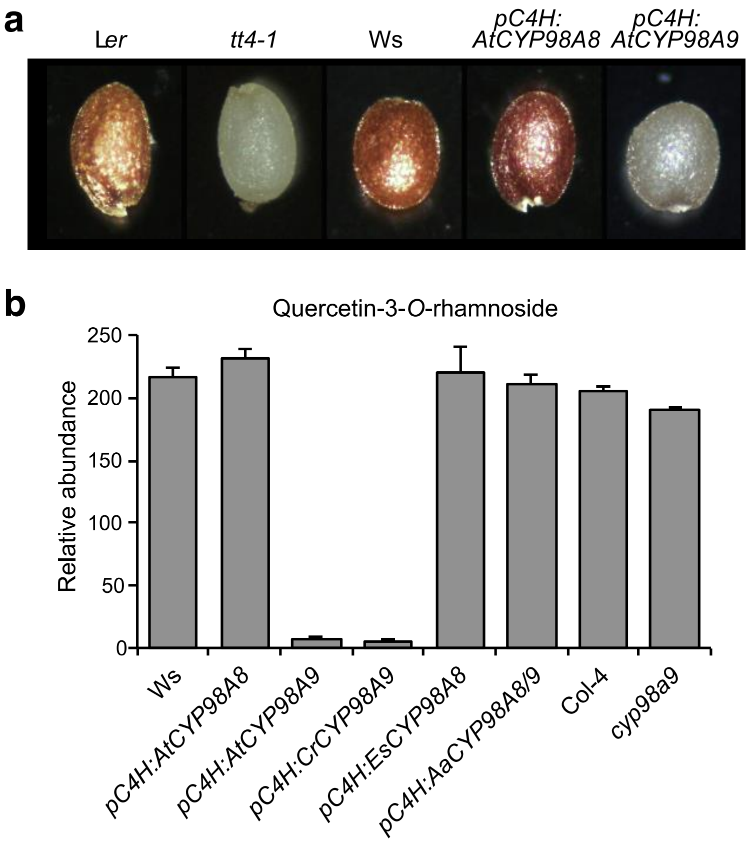

Supplementary Figure 6. Detection of flavonoids in seeds of the *A. thaliana* transgenic lines.

(**a**) Vanillin staining of tannins precursors confirmed the lack of tannins in the *pC4H:AtCYP98A9* line (Ws background) as in the *tt4-1* mutant (L*er* background). (**b**) Profiling of the seed soluble phenolics shows that lineage I *CYP98A9s* ectopic expression only leads to suppression of the major flavonol quercetin-3-*O*-rhamnoside. Error bars represent the SE from three independent determinations. Aa, *A. arabicum*; At, *A. thaliana*; Cr, *C. rubella*; Es: *E. salsugineum*. Col-4: wild-type Arabidopsis Columbia-4. Ws: wild-type Arabidopsis Wassilewskija.

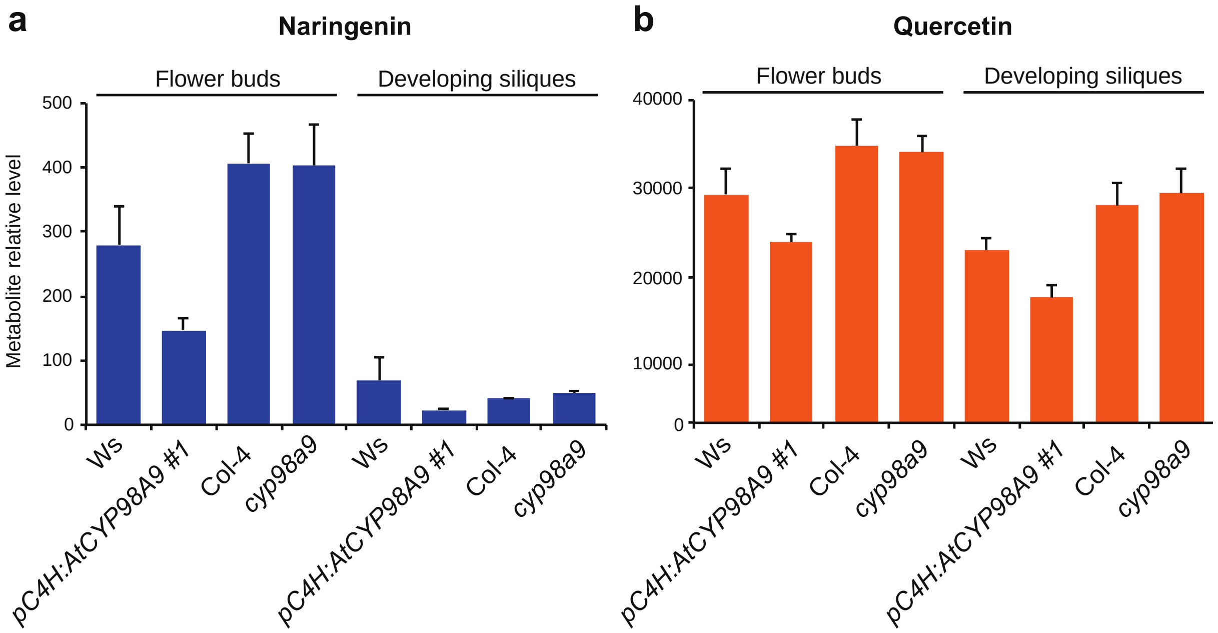

Supplementary Figure 7. Quantification of naringenin and quercetin aglycones in *A. thaliana* control and *CYP98A9* overexpressor and insertion mutant lines.

Relative amounts of (**a**) naringenin (blue) and (**b**) quercetin (orange) were quantified by LC-MS/MS after acid hydrolysis of extracts from flower buds and developing siliques at the globular seed stage. Error bars represent SE of three independent determinations.

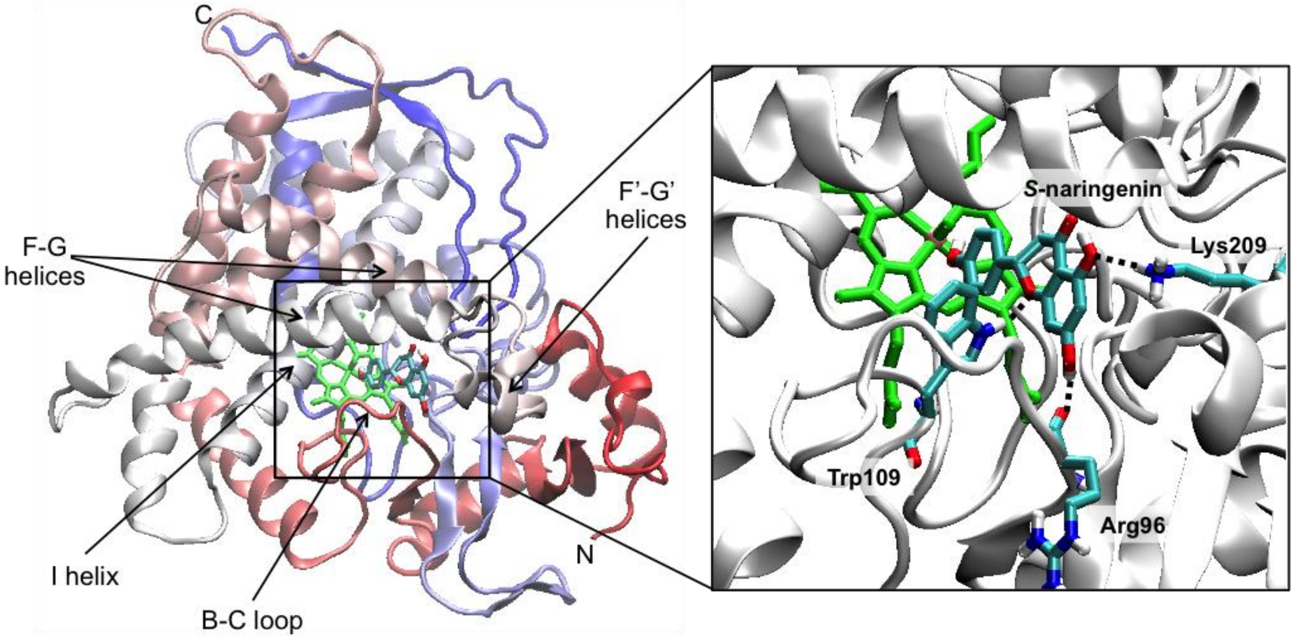

Supplementary Figure 8. **Alternative views of *S*-naringenin docking in the AtCYP98A9 active site.**

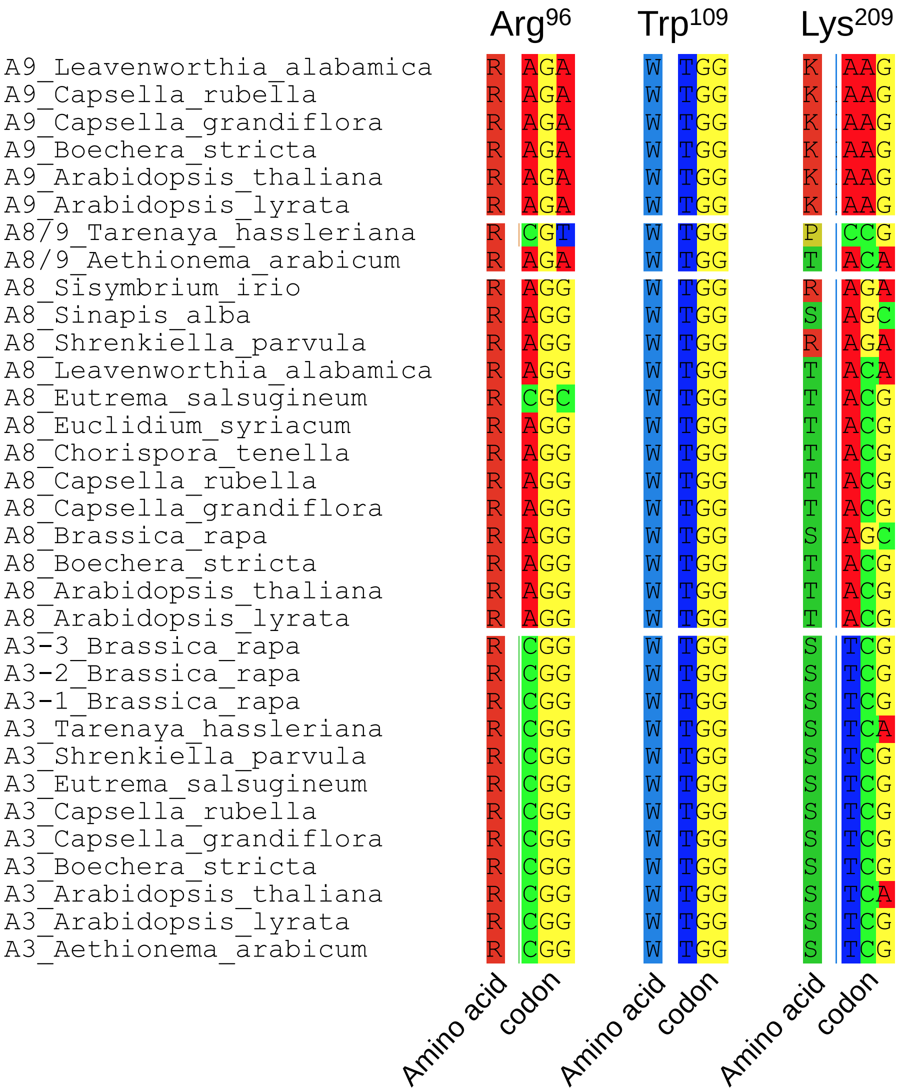

Supplementary Figure 9. **Alignments of the AtCYP98A9 Arg^{96}, Trp^{109} and Lys^{209} sites.**

Amino acid and codon alignments for the three CYP98A9 residues identified in *S*-naringenin docking experiments.

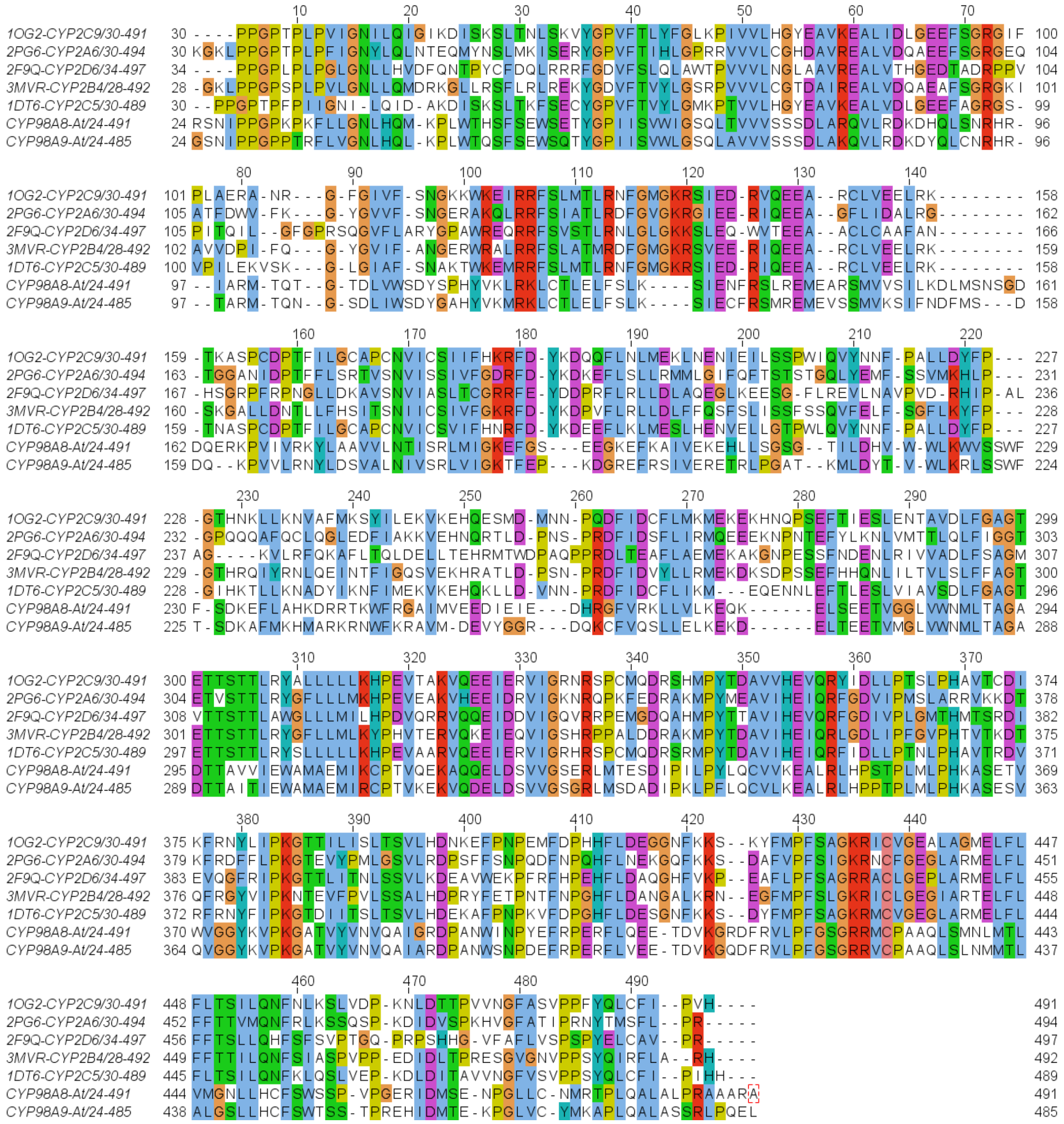

Supplementary Figure 10. Multiple proteins alignment used as input for homology modeling.

MAFFT (with iterative refinement method L-INS-i) was used to align the following templates: CYP2C9 (PDB 1OG2), CYP2A6 (PDB 2PG6), CYP2D6 (PDB 2F9Q), CYP2B4 (PDB 3MVR), and CYP2C5 (PDB 1DT6), together with the sequences of AtCYP98A8 (starting from Arg 24) and AtCYP98A9 (starting from Gly 24). Resulting alignment was the input for Modeller. The MAFFT-L-INS-I alignment has been corrected and validated by MUSTANG (MUltiple STructural AligNment AlGorithm).

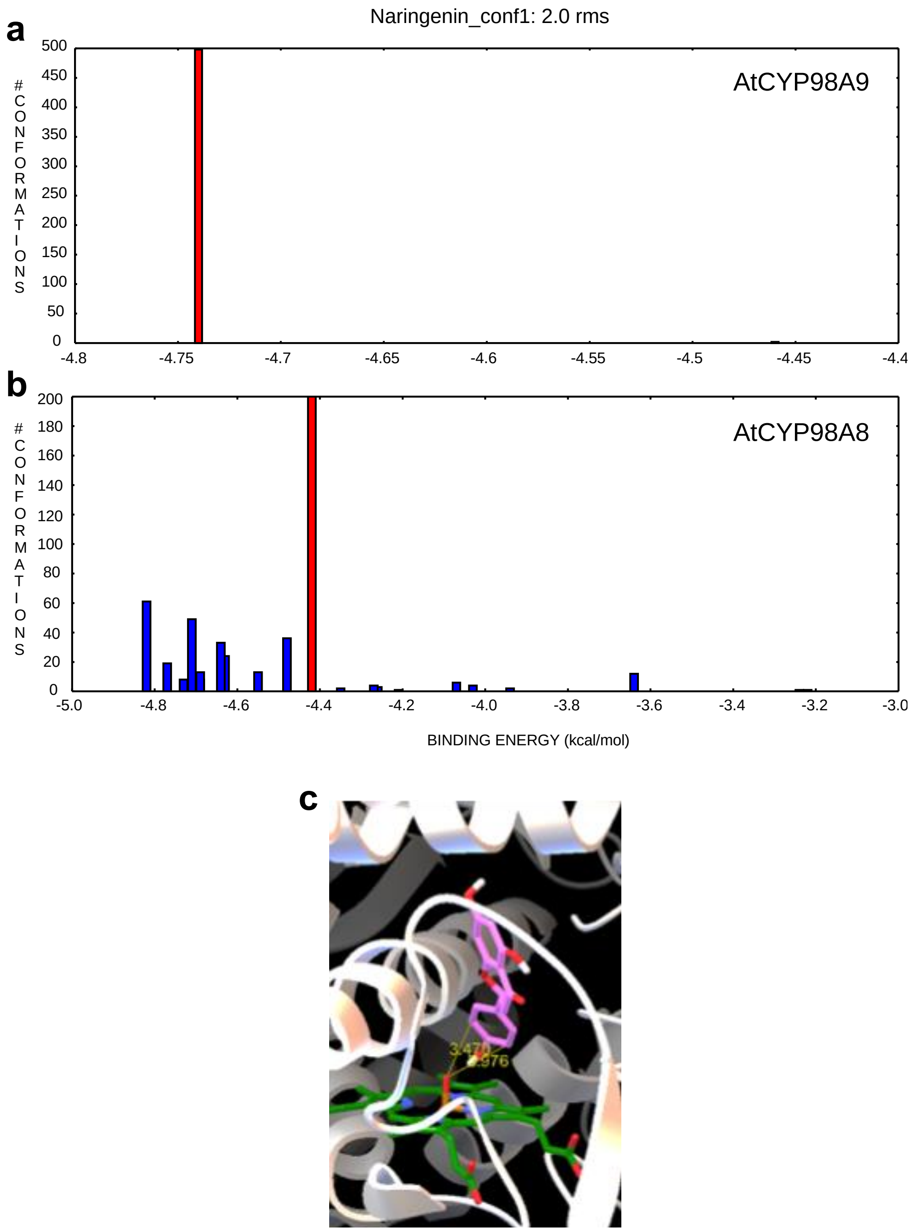

Supplementary Figure 11. **Clustering of 500 docking runs of one starting conformer of *S*-naringenin.**

Docking was performed with Autodock 4.2.6 on the rebuilt 3D models of AtCYP98A8 and AtCYP98A9 isoforms. The histograms of the docking scores (in kcal/mol) have been generated with RMSD tolerance of 2 Å for clustering. The distribution of poses between the two isoforms revealed highly dissimilar binding modes. The 500 calculated poses are found in an almost unique position (498/500) in (**a**) AtCYP98A9 (binding energy -4.74 kcal/mol, energy range of the cluster 0.7 kcal/mol), contrary to (**b**) AtCYP98A8 isoform, for which the poses are scattered in different orientations, and various positions and regions of the CYP active site, indicating reduced binding specificity. For AtCYP98A8, the lowest energy cluster (-4.73 kcal/mol) contains 61 similar poses of a reverse position of the ligand, with the double ring of naringenin oriented towards the iron-oxo group. The first cluster corresponding to a 3'-OH metabolism pose is found to be the 10th (in red, cluster of 200 poses, best binding energy -4.40 kcal/mol, energy range 0.5 kcal/mol), (**c**) with residues Trp109, Ile97 and Ser210 involved in H-bonding with naringenin. For AtCYP98A9, the bound conformation of naringenin is stabilized by Trp^{109}, Arg^{96}, and Lys^{209}, and is found for 498 out of 500 runs of the various naringenin conformers, with the same network of H-bonding interactions (**Fig. 5a**).

Supplementary Tables

Supplementary Table 1. List of CYP98A sequences ID and sources.

Sequence name	Sequence ID	Source
Aethionema arabicum A3	AA93G00030	CoGepedia
Aethionema arabicum A8/9	AA31G00492	CoGepedia
Arabidopsis lyrata A3	934902	Phytozome
Arabidopsis lyrata A8	476592	Phytozome
Arabidopsis lyrata A9	926836	Phytozome
Arabidopsis thaliana A3	At2g40890	Phytozome
Arabidopsis thaliana A8	At1g74540	Phytozome
Arabidopsis thaliana A9	At1g74550	Phytozome
Boechera stricta A3	Bostr.9345s0043	Phytozome
Boechera stricta A8	Bostr.3288s0100	Phytozome
Boechera stricta A9	Bostr.3288s0102	Phytozome
Brassica rapa A3-1	Bra004591	Phytozome
Brassica rapa A3-2	Bra000211	Phytozome
Brassica rapa A3-3	Bra016964	Phytozome
Brassica rapa A8	Bra003795	Phytozome
Capsella grandiflora A3	Cagra.1642s0055	Phytozome
Capsella grandiflora A8	Cagra.0402s0070	Phytozome
Capsella grandiflora A9	Cagra.0402s0071	Phytozome
Capsella rubella A3	Carubv10023039m	Phytozome
Capsella rubella A8	Carubv10020525m	Phytozome
Capsella rubella A9	scaffold_2:11600237..11601700	Phytozome
Chorispora tenella A8	KX754461	This study
Euclidium syriacum A8	KX754460	This study
Eutrema salsugineum A3	Thhalv10016538m	Phytozome
Eutrema salsugineum A8	Thhalv10019844m	Phytozome
Leavenworthia alabamica A8	LA_scaffold402_37	CoGepedia
Leavenworthia alabamica A9	LA_scaffold810_32	CoGepedia
Shrenkiella parvula A3	Tp4g23210	CoGepedia
Shrenkiella parvula A8	Tp5g29720	CoGepedia
Sinapis alba A8	scaffold-VMNH-2088376	1kP project
Sisymbrium irio A8	SI_scaffold242_307.mRNA1	CoGepedia
Tarenaya hassleriana A3	Th2v28411	CoGepedia
Tarenaya hassleriana A8/9	Th2v10286	CoGepedia

Supplementary Table 2. Results of likelihood ratio tests for the different d_N/d_S models.

The different d_N/d_S models were run either on the whole alignment (560 sites for Fitmodel analyses and 457 sites for PAML analyses) or on a trimmed alignment (404 sites, for both Fitmodel and PAML analyses). lnL: likelihood; df: degree of freedom.

	# parameters	lnL	df	p-value
**457 codons**				
**BranchModel**				
Test3 (4 ratio)		-16601.79404	1	
Test4 (4 ratio)		-16597.59446	1	3.8E-03
Test5 (5 ratio)/Test4		-16588.56815	1	2.1E-05
Test5 (5 ratio)/Test3		-16588.56815	1	2.7E-07
**BranchSite**				
Control2 (A9) fix w2=1		-16526.84164		
Test2 (A9)	4	-16526.84164	1	1.0E+00
Control3 (A8LI) fix w2=1		-16542.10764		
Test3 (A8LI)	4	-16542.03048	1	6.9E-01
Control4 (A8LII_III) fix w2=1		-16518.81450		
Test4 (A8LII_III)	4	-16518.81450	1	1.0E+00
**CladeModel**				
Control (M1a)	2	-16542.13271		
Test3 (A8 A9)	6	-16350.08619	4	<10E-308
Test5 (A8LI A9 A8LII_III)	7	-16354.14997	5	<10E-308
Test6 (A8LI+A9 A8LII_III)	6	-16361.85583	4	<10E-308
Test5 (A8LI A9 A8LII_III) / Test 3	7	-16354.14997	1	4.4E-03
Test5 (A8LI A9 A8LII_III) / Test 6	7	-16354.14997	1	8.6E-05
**560 codons**				
**FitModel**				
M2a No Switch		-19047.68739		
MX		-18853.12377	0/1	<10E-308
M2a		-18800.81296	0/1	<10E-308
**404 codons**				
**BranchModel**				
Test3 (4 ratio)		-13993.59631	1	
Test4 (4 ratio)		-13989.48893	1	4.2E-03
Test5 (5 ratio)/Test4		-13981.43364	1	6.0E-05
Test5 (5 ratio)/Test3		-13981.43364	1	8.1E-07
**BranchSite**				
Control2 (A9) fix w2=1		-13960.19083		

Test2 (A9)	4	-13960.19083	1	1.0E+00
Control3 (A8LI) fix w2=1		-13969.62255		
Test3 (A8LI)	4	-13969.44540	1	5.5E-01
Control4 (A8LII_III) fix w2=1		-13945.99915		
Test4 (A8LII_III)	4	-13945.99915	1	1.0E+00
**CladeModel**				
Control (M1a)	2	-13969.86568		
Test3 (A8 A9)	6	-13804.84842	4	<10E-308
Test5 (A8LI A9 A8LII_III)	7	-13805.06666	5	<10E-308
Test6 (A8LI+A9 A8LII_III)	6	-13811.82391	4	<10E-308
Test3 (A8 A9) / Test1	6	-13804.84842	1	
Test5 (A8LI A9 A8LII_III) / Test 3	7	-13805.06666	1	5.1E-01
Test5 (A8LI A9 A8LII_III) / Test 6	7	-13805.06666	1	2.4E-04
**FitModel**				
M2a No Switch		-13889.65632		
MX		-13884.56908	0/1	7.1E-04
M2a		-13841.69396	0/1	<10E-308

Supplementary Table 3. Results of the McDonald-Kreitman (MK) test.

CYP98A9 CDS from 848 *Arabidopsis thaliana* accessions were downloaded from the SALK Institute (http://signal.salk.edu/atg1001/index.php). *CYP98A9* CDS from *Leavenworthia alabamica* was used as the outgroup. There were 48 alleles in *Arabidopsis thaliana* accessions, which were used to implement a polarized MK using the program DNAsp (http://www.ub.edu/dnasp/).

============== McDonald and Kreitman Table ==============

Synonymous Substitutions

Fixed differences between species: 108   Polymorphic sites: 20

Nonsynonymous Substitutions:

Fixed differences between species: 90   Polymorphic sites: 38

Neutrality Index, NI: 2.280

Alfa value: -1.280

Fisher's exact test. P-value (two tailed): 0.010751*

Supplementary Table 4. Gene lists associated with the co-expression networks of AtCYP98A8 and AtCYP98A9.

Lists were obtained with PlaNet (http://aranet.mpimp-golm.mpg.de/).

Locus identifier	Gene description
CYP98A8 co-expression network	
AT5G60500	Undecaprenyl pyrophosphate synthetase family protein.
AT1G06990	GDSL-like Lipase/Acylhydrolase superfamily protein
AT2G03740	LEA domain-containing protein
AT1G23250	Caleosin-related family protein
AT3G57620	glyoxal oxidase-related protein
AT1G23600	Domain of unknown function DUF220
AT1G75920	GDSL-like Lipase/Acylhydrolase superfamily protein
AT1G30350	Pectin lyase-like superfamily protein
AT1G30020	Protein of unknown function, DUF538
AT3G45480	RING/U-box protein with C6HC-type zinc finger
AT2G03850	Late embryogenesis abundant protein (LEA) family protein
AT1G06250	alpha/beta-Hydrolases superfamily protein
AT1G33430	Galactosyltransferase family protein
AT3G23770	O-Glycosyl hydrolases family 17 protein
AT1G79780	Uncharacterised protein family (UPF0497)
AT1G02813	Protein of unknown function, DUF538
AT5G07230	Bifunctional inhibitor/lipid-transfer protein/seed storage 2S albumin superfamily protein
AT4G14815	Bifunctional inhibitor/lipid-transfer protein/seed storage 2S albumin superfamily protein
AT4G29250	HXXXD-type acyl-transferase family protein
AT1G23590	Domain of unknown function DUF220
AT5G48210	unknown protein
AT1G68875	unknown protein
AT1G23240	Caleosin-related family protein
AT1G26710	unknown protein;.
AT2G42180	unknown protein
AT5G16920	Fasciclin-like arabinogalactan family protein
AT1G28375	unknown protein
AT4G30040	Eukaryotic aspartyl protease family protein
AT1G20150	Subtilisin-like serine endopeptidase family protein
AT1G23520	Domain of unknown function (DUF220)
AT1G23570	Domain of unknown function (DUF220)
AT1G66850	Bifunctional inhibitor/lipid-transfer protein/seed storage 2S albumin superfamily protein
AT5G60510	Undecaprenyl pyrophosphate synthetase family protein
AT1G06170	basic helix-loop-helix (bHLH) DNA-binding superfamily protein
AT5G13380	Auxin-responsive GH3 family protein

AT2G18420	Encodes a Gibberellin-regulated GASA/GAST/Snakin family protein
AT1G23670	Domain of unknown function (DUF220)
AT5G62080	Bifunctional inhibitor/lipid-transfer protein/seed storage 2S albumin superfamily protein
AT1G36150	Bifunctional inhibitor/lipid-transfer protein/seed storage 2S albumin superfamily
AT1G23580	Domain of unknown function DUF220
AT5G61605	unknown protein
AT1G06260	Cysteine proteinases superfamily protein
AT1G01280	"CYTOCHROME P450, FAMILY 703, SUBFAMILY A, POLYPEPTIDE 2" (CYP703A2)
AT1G07340	SUGAR TRANSPORTER 2 (STP2)
AT1G08065	ALPHA CARBONIC ANHYDRASE 5 (ACA5)
AT1G13140	"CYTOCHROME P450, FAMILY 86, SUBFAMILY C, POLYPEPTIDE 3" (CYP86C3)
AT1G13150	"CYTOCHROME P450, FAMILY 86, SUBFAMILY C, POLYPEPTIDE 4" (CYP86C4)
AT1G15460	REQUIRES HIGH BORON 4 (BOR4)
AT1G26780	MYB DOMAIN PROTEIN 117 (MYB117)
AT1G28430	"CYTOCHROME P450, FAMILY 705, SUBFAMILY A, POLYPEPTIDE 24" (CYP705A24)
AT1G56360	PURPLE ACID PHOSPHATASE 6 (PAP6)
AT1G61070	LOW-MOLECULAR-WEIGHT CYSTEINE-RICH 66 (LCR66)
AT1G61110	NAC DOMAIN CONTAINING PROTEIN 25 (NAC025)
AT1G67990	TAPETUM-SPECIFIC METHYLTRANSFERASE 1 (ATTSM1)
AT1G69500	"CYTOCHROME P450, FAMILY 704, SUBFAMILY B, POLYPEPTIDE 1" (CYP704B1)
AT1G71160	3-KETOACYL-COA SYNTHASE 7 (KCS7)
AT1G74540	CYTOCHROME P450, FAMILY 98, SUBFAMILY A, POLYPEPTIDE 8 (CYP98A8)
AT1G74550	CYTOCHROME P450, FAMILY 98, SUBFAMILY A, POLYPEPTIDE 9 (CYP98A9)
AT1G75790	SKU5 SIMILAR 18 (sks18)
AT1G75910	EXTRACELLULAR LIPASE 4 (EXL4)
AT1G75930	EXTRACELLULAR LIPASE 6 (EXL6)
AT1G75940	BETA GLUCOSIDASE 20 (BGLU20)
AT2G19070	SPERMIDINE HYDROXYCINNAMOYL TRANSFERASE (SHT)
AT2G23800	GERANYLGERANYL PYROPHOSPHATE SYNTHASE 2 (GGPS2)
AT3G06100	NOD26-LIKE INTRINSIC PROTEIN 7;1 (NIP7;1)
AT3G11980	MALE STERILITY 2 (MS2)
AT3G13220	ATP-BINDING CASSETTE G26 (ABCG26)
AT3G15400	ANTHER 20 (ATA20)
AT3G25050	XYLOGLUCAN ENDOTRANSGLUCOSYLASE/HYDROLASE 3 (XTH3)
AT3G42960	TAPETUM 1 (ATA1)
AT3G51590	LIPID TRANSFER PROTEIN 12 (LTP12)
AT3G52160	3-KETOACYL-COA SYNTHASE 15 (KCS15)
AT3G52810	PURPLE ACID PHOSPHATASE 21 (PAP21)
AT4G14080	MATERNAL EFFECT EMBRYO ARREST 48 (MEE48)
AT4G27330	SPOROCYTELESS (SPL)

AT4G28090	SKU5 SIMILAR 10 (sks10)
AT4G28395	ANTHER 7 (A7)
AT4G34850	LESS ADHESIVE POLLEN 5 (LAP5)
AT5G07510	GLYCINE-RICH PROTEIN 14 (GRP14)
AT5G07520	GLYCINE-RICH PROTEIN 18 (GRP18)
AT5G07530	GLYCINE RICH PROTEIN 17 (GRP17)
AT5G07540	GLYCINE-RICH PROTEIN 16 (GRP16)
AT5G07550	GLYCINE-RICH PROTEIN 19 (GRP19)
AT5G07560	GLYCINE-RICH PROTEIN 20 (GRP20)
AT5G49070	3-KETOACYL-COA SYNTHASE 21 (KCS21)
AT5G53190	SWEET3
AT5G62320	MYB DOMAIN PROTEIN 99 (MYB99)
CYP98A9 co-expression network – Cluster A	
AT5G60500	Undecaprenyl pyrophosphate synthetase family protein.
AT5G60500	Undecaprenyl pyrophosphate synthetase family protein
AT1G06990	GDSL-like Lipase/Acylhydrolase superfamily protein
AT1G68875	unknown protein
AT1G23240	Caleosin-related family protein
AT5G52160	Bifunctional inhibitor/lipid-transfer protein/seed storage 2S albumin superfamily protein
AT3G23840	HXXXD-type acyl-transferase family protein
AT2G03740	late embryogenesis abundant domain-containing protein / LEA domain-containing protein
AT1G23250	Caleosin-related family protein
AT1G23600	Domain of unknown function DUF22
AT1G30350	Pectin lyase-like superfamily protein
AT1G23520	Domain of unknown function (DUF220
AT1G30020	Protein of unknown function, DUF538
AT1G23570	Domain of unknown function (DUF220)
AT3G45480	RING/U-box protein with C6HC-type zinc finger
AT1G06250	alpha/beta-Hydrolases superfamily protein
AT1G33430	Galactosyltransferase family protein
AT1G06170	basic helix-loop-helix (bHLH) DNA-binding superfamily protein
AT5G60510	Undecaprenyl pyrophosphate synthetase family protein
AT1G79780	Uncharacterised protein family (UPF0497)
AT3G23770	O-Glycosyl hydrolases family 17 protein
AT2G18420	Encodes a Gibberellin-regulated GASA/GAST/Snakin family protein
AT3G42850	Mevalonate/galactokinase family protein
AT1G23580	Domain of unknown function DUF220
AT5G61605	unknown protein
AT1G06260	Cysteine proteinases superfamily protein
AT1G23690	Domain of unknown function DUF22

AT1G01280	"CYTOCHROME P450, FAMILY 703, SUBFAMILY A, POLYPEPTIDE 2" (CYP703A2)
AT1G28430	"CYTOCHROME P450, FAMILY 705, SUBFAMILY A, POLYPEPTIDE 24" (CYP705A24)
AT1G13140	"CYTOCHROME P450, FAMILY 86, SUBFAMILY C, POLYPEPTIDE 3" (CYP86C3)
AT1G13150	"CYTOCHROME P450, FAMILY 86, SUBFAMILY C, POLYPEPTIDE 4" (CYP86C4)
AT3G52160	3-KETOACYL-COA SYNTHASE 15 (KCS15)
AT1G71160	3-KETOACYL-COA SYNTHASE 7 (KCS7)
AT2G16910	ABORTED MICROSPORES (AMS)
AT1G08065	ALPHA CARBONIC ANHYDRASE 5 (ACA5)
AT4G28700	AMMONIUM TRANSPORTER 1;4 (AMT1;4)
AT3G26940	CONSTITUTIVE DIFFERENTIAL GROWTH 1 (CDG1)
AT1G74540	CYTOCHROME P450, FAMILY 98, SUBFAMILY A, POLYPEPTIDE 8 (CYP98A8)
AT2G23800	GERANYLGERANYL PYROPHOSPHATE SYNTHASE 2 (GGPS2)
AT5G07530	GLYCINE RICH PROTEIN 17 (GRP17)
AT5G07510	GLYCINE-RICH PROTEIN 14 (GRP14)
AT5G07540	GLYCINE-RICH PROTEIN 16 (GRP16)
AT5G07520	GLYCINE-RICH PROTEIN 18 (GRP18)
AT1G61070	LOW-MOLECULAR-WEIGHT CYSTEINE-RICH 66 (LCR66)
AT5G53510	OLIGOPEPTIDE TRANSPORTER 9 (OPT9)
AT3G52810	PURPLE ACID PHOSPHATASE 21 (PAP21)
AT5G36150	PUTATIVE PENTACYCLIC TRITERPENE SYNTHASE 3 (PEN3)
AT5G55590	QUARTET 1 (QRT1)
AT2G19070	SPERMIDINE HYDROXYCINNAMOYL TRANSFERASE (SHT)
AT3G42960	TAPETUM 1 (ATA1)
CYP98A9 co-expression network – Cluster B	
AT5G60500	Undecaprenyl pyrophosphate synthetase family protein.
AT5G12460	Protein of unknown function (DUF604)
AT5G13600	Phototropic-responsive NPH3 family protein
AT5G07260	START (StAR-related lipid-transfer) lipid-binding domain
AT5G03810	GDSL-like Lipase/Acylhydrolase family protein
AT1G04380	encodes a protein similar to a 2-oxoglutarate-dependent dioxygenase
AT3G02590	Fatty acid hydroxylase superfamily protein
AT1G50650	Stigma-specific Stig1 family protein
AT1G69860	Major facilitator superfamily protein
AT1G23510	unknown protein
AT5G03820	GDSL-like Lipase/Acylhydrolase family protein
AT4G10490	2-oxoglutarate (2OG) and Fe(II)-dependent oxygenase superfamily protein
AT4G33600	unknown protein
AT2G14690	Encodes a putative glycosyl hydrolase family 10 protein (xylanase).
AT3G46240	Receptor protein kinase-related
AT3G29300	unknown protein

AT3G18180	Glycosyltransferase family 61 protein
AT5G38180	Bifunctional inhibitor/lipid-transfer protein/seed storage 2S albumin superfamily protein
AT1G64800	Sequence-specific DNA binding transcription factors
AT3G11180	2-oxoglutarate (2OG) and Fe(II)-dependent oxygenase superfamily protein
AT1G54955	unknown protein
AT1G71250	GDSL-like Lipase/Acylhydrolase superfamily protein
AT3G20950	"CYTOCHROME P450, FAMILY 705, SUBFAMILY A, POLYPEPTIDE 32" (CYP705A32)
AT3G52970	"CYTOCHROME P450, FAMILY 76, SUBFAMILY G, POLYPEPTIDE 1" (CYP76G1)
AT1G62340	ABNORMAL LEAF-SHAPE 1 (ALE1)
AT5G27200	ACYL CARRIER PROTEIN 5 (ACP5)
AT5G10220	ANNEXIN 6 (ANN6)
AT3G58740	CITRATE SYNTHASE 1 (CSY1)
AT5G24900	CYTOCHROME P450, FAMILY 714, SUBFAMILY A, POLYPEPTIDE 2 (CYP714A2)
AT5G57920	EARLY NODULIN-LIKE PROTEIN 10 (ENODL10)
AT4G28365	EARLY NODULIN-LIKE PROTEIN 3 (ENODL3)
AT4G32490	EARLY NODULIN-LIKE PROTEIN 4 (ENODL4)
AT1G71120	GDSL-MOTIF LIPASE/HYDROLASE 6 (GLIP6)
AT4G10640	IQ-DOMAIN 16 (IQD16)
AT3G27785	MYB DOMAIN PROTEIN 118 (MYB118)
AT3G19050	PHRAGMOPLAST ORIENTING KINESIN 2 (POK2)
AT4G33330	PLANT GLYCOGENIN-LIKE STARCH INITIATION PROTEIN 3 (PGSIP3)
AT3G08900	REVERSIBLY GLYCOSYLATED POLYPEPTIDE 3 (RGP3)
AT1G14750	SOLO DANCERS (SDS)
AT5G59590	UDP-GLUCOSYL TRANSFERASE 76E2 (UGT76E2)

Supplementary Table 5. ***A. thaliana*** **seed flavonols profiling in transgenic lines.**

Flavonols relative amounts in seeds of *pC4H:AtCYP98A8* and *pC4H:AtCYP98A9* lines were determined by UPLC-MS/MS. For transgenic lines, results are the mean ± SE of measurements made on seeds samples from three independent lines. For wild type (Ws), results are the mean ± SE of measurements made on three independent seeds samples. n.d., not detected; Rha, rhamnoside; Glu, glucoside.

A. thaliana **flavonols**	**Ws**	***pC4H:AtCYP98A8***	***pC4H:AtCYP98A9***
Kaempferol-3-*O*-Rha	0.15 ±0.07	0.30 ±0.02	n.d.
Kaempferol-3-*O*-Glu-7-*O*-Rha	2.94 ±0.62	2.12 ±0.06	2.03 ±0.10
Kaempferol-3-*O*-Rha-7-*O*-Rha	2.40 ±0.37	1.90 ±0.05	1.78 ±0.13
Quercetin-3-*O*-Rha	9.54 ±0.97	15.45 ±0.58	0.30 ±0.05
Quercetin-3-*O*-Glu-7-*O*-Rha	5.27 ±0.93	2.50 ±0.19	3.09 ±0.18
Quercetin-3-*O*-Rha-7-*O*-Rha	5.35 ±0.43	3.57 ±0.13	2.96 ±0.09
Isorhamnetin-3-*O*-Rha	0.71 ±0.12	0.68 ±0.06	0.15 ±0.03
Isorhamnetin-3-*O*-Glu-7-*O*-Rha	2.11 ±0.44	1.02 ±0.03	1.76 ±0.03
Isorhamnetin-3-*O*-Rha-7-*O*-Rha	1.38 ±0.25	0.76 ±0.05	0.89 ±0.02

Supplementary Table 6. List of primers used in the study.

Gateway attB and USER sequences are underlined.

Primer name	Sequence (5’ > 3’)
T-DNA mutant genotyping	
cyp98a9_KO_LP	GACGATGACGATGAAGAGGAG
cyp98a9_KO_RP	TTACACCGTTTGGCTTAAACG
SK_T-DNA	ATACGACGGATCGTAATTTGTCG
qPCR analysis	
AtCYP98A8_qPCR_F	GCCATCGTTGAGAAAGAACATCTT
AtCYP98A8_qPCR_R	AACCCATTTGAGCCACCAGAC
AtCYP98A9_qPCR_F	CACCGTTTGGCTTAAAC
AtCYP98A9_qPCR_R	CCGAGCCATGTGCTTCAT
AtSAND_qPCR_F	AACTCTTATGCAGCATTTGATCCACT
AtSAND_qPCR_R	TGATTGCATATCTTTATCGCCATC
CDS gateway cloning	
AtCYP98A8_AttB1	GGGGACAAGTTTGTACAAAAAAGCAGGCTTCATGATTATATATCTAATTTCATTACTCCCT
AtCYP98A8_AttB2	GGGGACCACTTTGTACAAGAAAGCTGGGTCTTAATCTAAAGGTAAAGGTATTGCTC
AtCYP98A9_AttB1	GGGGACAAGTTTGTACAAAAAAGCAGGCTTCATGGATTTATTACTCATATCCTTAACC
AtCYP98A9_AttB2	GGGGACCACTTTGTACAAGAAAGCTGGGTCTTAAAGGTATAACTCTTGTGGCAG
CrCYP98A9_AttB1	GGGGACAAGTTTGTACAAAAAAGCAGGCTTCATGGATTTATTACTGATTACAC
CrCYP98A9_AttB2	GGGGACCACTTTGTACAAGAAAGCTGGGTCTTAAAGGTATAACTCTTGTGGC
EsCYP98A8_AttB1	GGGGACAAGTTTGTACAAAAAAGCAGGCTTCATGGTTCTATATGTAATTTCTC
EsCYP98A8_AttB2	GGGGACCACTTTGTACAAGAAAGCTGGGTCTCACAAGTTATAAGGTATAAC
AaCYP98A8/9_AttB1	GGGGACAAGTTTGTACAAAAAAGCAGGCTTCATGGTTCCATTACTGATTTCGC
AaCYP98A8/9_AttB2	GGGGACCACTTTGTACAAGAAAGCTGGGTCTTACCTATCTATTTCAGCTAAAC
CDS USER cloning	
EsCYP98A8U_F	GGCTTAAUATGGTTCTATATGTAATTTCTC
EsCYP98A8U_R	GGTTTAAUTCACAAGTTATAAGGTATAAC
AaCYP98A8/9U_F	GGCTTAAUATGGTTCCATTACTGATTTCGCTAAC
AaCYP98A8/9U_F	GGTTTAAUTTACCTATCTATTTCAGCTAAACTTAAAG
ThCYP98A8/9U_F	GGCTTAAUATGCTCCTGCTGCTTATTTCATC
ThCYP98A8/9U_F	GGCTTAAUATGCTCCTGCTGCTTATTTCATC
Degenerate TAIL PCR (used in combination with LAD primers)	
CYP_5to3_TAIL_outer_alltaxa	ATCCAACATACCKCCRGGACC
CYP_5to3_TAIL_inner_alltaxa	GGYCCHATMATATCDGTRTGG
CYP_3to5_TAIL_outer_alltaxa	TCCAHGAGAARCAWTGCAAC
CYP_3to5_TAIL_inner_alltaxa	CYGAYCCRAAHGGAAGAAG
Species specific TAIL PCR (used in combination with LAD primers)	
Esyria_TAIL2_5to3_inner	AGAGTGAGGAAGATGATTGTCCC

Esyria_TAIL2_3to5_outer	GTTTCACATAGTGTGGATTGTAATCGG
Esyria_TAIL2_3to5_inner	CGACCCTGTGTCGGTTAGAC
Ct_TAIL2_5to3_outer	AGATGTAGCTATGGCGAAAGATTAAAATG
Ct_TAIL2_5to3_inner	ATTGCTTAGAATTGAGAACCTCATAACAC
Ct_TAIL2_3to5_outer	AAGTTCGAGTGTACAGAGTTTCCGC
Ct_TAIL2_3to5_inner	CCTTGCGATCCTGTGTCGG
Amplification of species-specific CYP98A8/9 locus	
Ct_fragment_F	GGGAGTGGGTCATTCGGTTTC
Ct_fragment_R	TGGAAATCTCCTCTTGTCCAGG
Es_fragment_F	GCGGCAAGGGTTAAGTGTAATG
Es_FullFrag_R	CGTGTACGTTTGGTTTTCTGCC

Supplementary Note 1

Detailed results of the d_N/d_S analysis
(16 species; 33 sequences)

Whole Alignment (457 codons used in the analysis):

PAML

Branch Model:

```
Test3:
model=2 NSites=0              wb     wf1 (A3)    wf2 (A8)    wf3 (A9)
w (dN/dS) for branches:  0.20503  0.08001     0.18244     0.22458
lnL0 = -18031.432706
lnL  = -16601.794038

Test4:
model=2 NSites=0              wb     wf1 (A3) wf2 (A8LI+A9) wf3 (A8LII_III)
w (dN/dS) for branches:  0.24444   0.08008     0.16199     0.20169
lnL0 = -18035.691187
lnL  = -16597.594460

Test5:
model=2 NSites=0              wb     wf1 (A3)    wf2 (A8LI) wf3 (A9) wf4
(A8LII_III)
w (dN/dS) for branches:  0.24090   0.08006     0.11401     0.22126     0.20223
lnL0 = -18043.297535
lnL  = -16588.568145

---------
```

Clade Model:

```
Control: w0 w1    M1A from site model model=0 NSites=1

dN/dS (w) for site classes (K=2)

proportions
p:    0.86775  0.13225
        w0        w1
w:    0.12137  1.00000

lnL0 = -17473.026637
lnL  = -16542.132707


Test3 w0 w1 w2 (A8) w3 (A9)    model=3 NSites=2

dN/dS (w) for site classes (K=3)

site class              0          1          2
proportion          0.53672    0.01010    0.45317
                         w0                    w1
branch type 0:      0.03556    1.00000    0.20660
                         w0                    w2 (A8)
branch type 1:      0.03556    1.00000    0.36956
                         w0                    w3 (A9)
branch type 2:      0.03556    1.00000    0.42991

lnL0 = -17243.101151
lnL  = -16350.086194
```

Test6 w0 w1 w2 (A8LI+A9) w3 (A8LII_III)   model=3 NSites=2

dN/dS (w) for site classes (K=3)

site class	0	1	2
proportion	0.54995	0.00882	0.44124
	**w0**		**w1**
branch type 0:	0.03853	1.00000	0.23962
	**w0**		**w2 (A8LI+A9)**
branch type 1:	0.03853	1.00000	0.32450
	**w0**		**w3 (A8LII_III)**
branch type 2:	0.03853	1.00000	0.41842

lnL0 = -17243.186246
lnL = -16361.855829

Test5 w0 w1 w2 (A8LI) w3 (A9) w4 (A8LII_III)   model=3 NSites=2

dN/dS (w) for site classes (K=3)

site class	0	1	2
proportion	0.52184	0.01158	0.46658
	**w0**		**w1**
branch type 0:	0.03425	1.00000	0.22857
	**w0**		**w2 (A8LI)**
branch type 1:	0.03425	1.00000	0.21602
	**w0**		**w3 (A9)**
branch type 2:	0.03425	1.00000	0.43443
	**w0**		**w4 (A8LII_III)**
branch type 3:	0.03425	1.00000	0.39346

lnL0 = -17245.676264
lnL = -16354.149966

<u>Branch Site:</u>

Control   M2A model=2 NSites=2 fixed omega=1

Control2 A9
foreground A9

dN/dS (w) for site classes (K=4)

site class	0	1	2a	2b
proportion	0.77134	0.11445	0.09945	0.01476
background w	0.11295	1.00000	0.11295	1.00000
foreground w	0.11295	1.00000	1.00000	1.00000

lnL0 = -17071.283119
lnL = -16526.841641

Control3 A8LI
foreground A8LI

dN/dS (w) for site classes (K=4)

site class	0	1	2a	2b
proportion	0.86624	0.13124	0.00219	0.00033
background w	0.12124	1.00000	0.12124	1.00000
foreground w	0.12124	1.00000	1.00000	1.00000

```
lnL0 = -17085.861311
lnL  = -16542.107635
```

Control4 A8LII_III
foreground A8LII_III

```
dN/dS (w) for site classes (K=4)

site class             0        1        2a       2b
proportion       0.78944  0.09322  0.10495  0.01239
background w     0.11199  1.00000  0.11199  1.00000
foreground w     0.11199  1.00000  1.00000  1.00000

lnL0 = -17081.346438
lnL  = -16518.814503
```

Test2 w0 w1 w2 (A9)   M2A model=2 NSites=2
foreground w2 A9

```
dN/dS (w) for site classes (K=4)

site class             0        1        2a       2b
proportion       0.77134  0.11445  0.09945  0.01476
background w     0.11295  1.00000  0.11295  1.00000
foreground w     0.11295  1.00000  1.00000  1.00000

lnL0 = -17080.625929
lnL  = -16526.841641
```

Test3 w0 w1 w2 (A8LI) M2A model=2 NSites=2
foreground w2 A8LI

```
dN/dS (w) for site classes (K=4)

site class             0        1        2a       2b
proportion       0.86658  0.13060  0.00245  0.00037
background w     0.12121  1.00000  0.12121  1.00000
foreground w     0.12121  1.00000  1.76221  1.76221

lnL0 = -17101.191579
lnL  = -16542.030475
```

Test4 w0 w1 w2 (A8LII_III) M2A model=2 NSites=2
foreground w2 A8LII_III

```
dN/dS (w) for site classes (K=4)

site class             0        1        2a       2b
proportion       0.78944  0.09322  0.10495  0.01239
background w     0.11199  1.00000  0.11199  1.00000
foreground w     0.11199  1.00000  1.00000  1.00000

lnL0 = -17095.520560
lnL  = -16518.814503

---------
```

FitModel

Whole Alignment (560 codons (total number) used in the analysis):

Control: no Switch

Model of codon substitution : M2a

Discrete gamma model : No

Transition/transversion ratio : 1.932

```
. p1 =          0.817649
. p2 =          0.181120
. p3 =          0.001232
. w1 =          0.126670
. w2 =          1.000000
. w3 =          2.589530
```

. log likelihood = -19047.687394

Model MX+S1

```
. p0 = 0.804251 | w0 = 0.038332
. p1 = 0.192648 | w1 = 1.000011
. p2 = 0.003101 | w2 = 3.476046
. delta = 0.340398
. R01 = 1.077012
. R02 = 1.077012
. R12 = 1.077012
```

```
site 175     0.164118   0.522028   0.313854
```

. log likelihood = -18853.123769

Model M2a+S1

```
. p0 = 0.579960 | w0 = 0.012966
. p1 = 0.340699 | w1 = 0.323848
. p2 = 0.079341 | w2 = 1.000000
. delta = 0.229046
. R01 = 0.423160
. R02 = 0.423160
. R12 = 0.423160
```

. log likelihood = -18800.812959

GBlocks (404 codons used in the analysis):

PAML

Branch Model:

Test3:

**model=2 NSites=0**	**wb**	**wf1 (A3)**	**wf2 (A8)**	**wf3 (A9)**
w (dN/dS) for branches:	0.17346	0.06961	0.16170	0.19354

lnL0 = -15431.232690
lnL = -13993.596314

Test4:

**model=2 NSites=0**	**wb**	**wf1 (A3)**	**wf2 (A8LI+A9)**	**wf3 (A8LII_III)**
w (dN/dS) for branches:	0.20678	0.06967	0.13830	0.18354

lnL0 = -15435.764285
lnL = -13989.488925

Test5:
model=2 NSites=0 wb wf1 (A3) wf2 (A8LI) wf3 (A9) wf4 (A8LII_III)

w (dN/dS) for branches: 0.20410 0.06964 0.09470 0.19127 0.18398
lnL0 = -15443.201435
lnL = -13981.433644

<u>Clade Model:</u>

Control:
M1A from site model model=0 NSites=1

dN/dS (w) for site classes (K=2)

p:	0.90770	0.09230
	**w0**	**w1**
w:	0.11159	1.00000

lnL0 = -14971.352051
lnL = -13969.865682

Test3 w0 w1 w2 (A8) w3 (A9) model=3 NSites=2

dN/dS (w) for site classes (K=3)

site class	0	1	2
proportion	0.55421	0.00542	0.44036
	**w0**		**w1**
branch type 0:	0.03094	1.00000	0.18957
	**w0**		**w2 (A8)**
branch type 1:	0.03094	1.00000	0.33602
	**w0**		**w3 (A9)**
branch type 2:	0.03094	1.00000	0.37812

lnL0 = -14791.066150
lnL = -13804.848420

Test6 w0 w1 w2 (A8LI+A9) w3 (A8LII_III) model=3 NSites=2

dN/dS (w) for site classes (K=3)

site class	0	1	2
proportion	0.56392	0.00606	0.43001
	**w0**		**w1**
branch type 0:	0.03305	1.00000	0.21432
	**w0**		**w2 (A8LI+A9)**
branch type 1:	0.03305	1.00000	0.28014
	**w0**		**w3 (A8LII_III)**
branch type 2:	0.03305	1.00000	0.39100

lnL0 = -14791.113850
lnL = -13811.823912

Test5 w0 w1 w2 (A8LI) w3 (A9) w4 (A8LII_III) model=3 NSites=2

dN/dS (w) for site classes (K=3)

site class	0	1	2
proportion	0.53593	0.00737	0.45671
	**w0**		**w1**
branch type 0:	0.02911	1.00000	0.20469

	w0		w2 (A8LI)
branch type 1:	0.02911	1.00000	0.18242
	**w0**		**w3 (A9)**
branch type 2:	0.02911	1.00000	0.37956
	**w0**		**w4 (A8LII_III)**
branch type 3:	0.02911	1.00000	0.36920

lnL0 = -14793.704778
lnL = -13805.066656

<u>Branch Site</u>

Control: w0 w1 w2=1 M2A model=2 NSites=2 fixed omega=1

Control2 A9
foreground A9

dN/dS (w) for site classes (K=4)

site class	0	1	2a	2b
proportion	0.83975	0.08152	0.07176	0.00697
background w	0.10592	1.00000	0.10592	1.00000
foreground w	0.10592	1.00000	1.00000	1.00000

lnL0 = -14623.994095
lnL = -13960.190829

Control3 A8LI
foreground A8LI

dN/dS (w) for site classes (K=4)

site class	0	1	2a	2b
proportion	0.90310	0.08912	0.00708	0.00070
background w	0.11121	1.00000	0.11121	1.00000
foreground w	0.11121	1.00000	1.00000	1.00000

lnL0 = -14633.749979
lnL = -13969.622551

Control4 A8LII_III
foreground A8LII_III

dN/dS (w) for site classes (K=4)

site class	0	1	2a	2b
proportion	0.82254	0.05949	0.11001	0.00796
background w	0.10172	1.00000	0.10172	1.00000
foreground w	0.10172	1.00000	1.00000	1.00000

lnL0 = -14633.316635
lnL = -13945.999147

Test2 w0 w1 w2 (A9) M2A model=2 NSites=2
foreground w2 (A9)

dN/dS (w) for site classes (K=4)

site class	0	1	2a	2b
proportion	0.83975	0.08152	0.07176	0.00697
background w	0.10592	1.00000	0.10592	1.00000

```
foreground w      0.10592  1.00000  1.00000  1.00000

lnL0 = -14634.076941
lnL  = -13960.190829
```

Test3 w0 w1 w2 (A8LI) M2A model=2 NSites=2
foreground w2 (A8LI)

```
dN/dS (w) for site classes (K=4)

site class             0        1        2a       2b
proportion        0.90506  0.08868  0.00570  0.00056
background w      0.11127  1.00000  0.11127  1.00000
foreground w      0.11127  1.00000  1.68871  1.68871

lnL0 = -14647.575174
lnL  = -13969.445402
```

Test4 w0 w1 w2 (A8LII_III) M2A model=2 NSites=2
foreground w2 (A8LII_III)

```
dN/dS (w) for site classes (K=4)

site class             0        1        2a       2b
proportion        0.82254  0.05949  0.11001  0.00796
background w      0.10172  1.00000  0.10172  1.00000
foreground w      0.10172  1.00000  1.00000  1.00000

lnL0 = -14649.242961
lnL  = -13945.999147

---------
```

GBlocks (404 codons used in the analysis)

FitModel

Control: M2a no Switch

```
Model of codon substitution : M2a

Discrete gamma model : No

. Transition/transversion ratio : 1.773

. p1 =            0.557537
. p2 =            0.431086
. p3 =            0.011377
. w1 =            0.034028
. w2 =            0.267033
. w3 =            1.000000

. log likelihood = -13889.656315
```

Model MX+S1

```
. p0 = 0.856367 | w0 = 0.031257
. p1 = 0.143590 | w1 = 1.000016
. p2 = 0.000043 | w2 = 1.500024
. delta = 0.405631
. R01 = 1.648789
. R02 = 1.648789
. R12 = 1.648789
```

. log likelihood = -13884.569083

Model M2a+S1

. p0 = 0.655803 | w0 = 0.012682
. p1 = 0.311965 | w1 = 0.334046
. p2 = 0.032232 | w2 = 1.000000
. delta = 0.256107
. R01 = 0.543104
. R02 = 0.543104
. R12 = 0.543104

. log likelihood = -13841.693963
